# Histidine Phosphorylation: Protein Kinases and Phosphatases

**DOI:** 10.3390/ijms25147975

**Published:** 2024-07-21

**Authors:** Jia Ning, Margaux Sala, Jeffrey Reina, Rajasree Kalagiri, Tony Hunter, Brandon S. McCullough

**Affiliations:** Molecular and Cell Biology Laboratory, Salk Institute for Biological Studies, La Jolla, CA 92037, USA; msala@salk.edu (M.S.); jreina@salk.edu (J.R.); rkalagiri@salk.edu (R.K.); hunter@salk.edu (T.H.)

**Keywords:** NME, histidine kinase, phosphorylation, histidine phosphatase

## Abstract

Phosphohistidine (pHis) is a reversible protein post-translational modification (PTM) that is currently poorly understood. The P-N bond in pHis is heat and acid-sensitive, making it more challenging to study than the canonical phosphoamino acids pSer, pThr, and pTyr. As advancements in the development of tools to study pHis have been made, the roles of pHis in cells are slowly being revealed. To date, a handful of enzymes responsible for controlling this modification have been identified, including the histidine kinases NME1 and NME2, as well as the phosphohistidine phosphatases PHPT1, LHPP, and PGAM5. These tools have also identified the substrates of these enzymes, granting new insights into previously unknown regulatory mechanisms. Here, we discuss the cellular function of pHis and how it is regulated on known pHis-containing proteins, as well as cellular mechanisms that regulate the activity of the pHis kinases and phosphatases themselves. We further discuss the role of the pHis kinases and phosphatases as potential tumor promoters or suppressors. Finally, we give an overview of various tools and methods currently used to study pHis biology. Given their breadth of functions, unraveling the role of pHis in mammalian systems promises radical new insights into existing and unexplored areas of cell biology.

## 1. Introduction to Histidine Phosphorylation

One of the key drivers of cell signaling is protein phosphorylation, an important post-translational modification (PTM) that is reversible through the activity of two families of enzymes, the kinases and the phosphatases, which add and remove phosphate molecules from target proteins, respectively. Nine proteinaceous amino acids have been reported to be phosphorylated, including serine, threonine, tyrosine, aspartate, cysteine, glutamate, arginine, and lysine. The ninth residue, histidine, can undergo phosphorylation through modification of its side chain imidazole ring. Upon phosphorylation of the imidazole ring, histidine is capable of forming two isomers, 1-phosphohistidine (1-pHis) and 3-phosphohistidine (3-pHis) [1]. While both forms have been observed in biological samples, 3-pHis is more stable chemically compared to 1-pHis [2]. Unlike the more commonly known phosphoamino acids pSer, pThr, and pTyr, which are acid stable and relatively heat insensitive, phosphohistidine (pHis) is highly acid sensitive and thermally labile, which creates unique challenges in studying this phosphoamino acid.

pHis was first discovered in 1962 by Paul D. Boyer on succinyl coenzyme A synthetase (SCS) in bovine liver lysates [3] and was later found to be present across all kingdoms of life. Subsequent studies have uncovered the role of pHis primarily in prokaryotes, where it acts as a signaling molecule in two-component signal transduction systems. As the name suggests, two-component signal transduction systems utilize two main proteins: membrane-bound His kinases and cytoplasmic response regulator proteins. In the presence of external stimuli, the phosphohistidine kinase (His kinase) becomes autophosphorylated on its catalytic histidine residue. This phosphate is then transferred from the histidine to an aspartate residue in the response regulator protein, which is usually a transcription factor. As a result, the transcription factor initiates a change in the expression of genes involved in virulence, motility, nutrient uptake, etc., in accordance with the signal received at the membrane [4]. Yeast and plants also adopted more complex two-component systems called multicomponent signal transduction. In response to a signal, the His kinase becomes activated, and the phosphate shuttles between several His kinases and response regulator proteins before a response is generated. Other than its role as a signaling molecule, pHis is known to have a key function as an enzyme intermediate in enzymes such as nucleoside diphosphate kinase A (NME1), ATP citrate lyase (ACLY), succinyl-CoA synthetase (SCSα), and phosphoglycerate mutase 1 (PGAM1) [1]. As its pKa is close to physiological pH, histidine can act either as a nucleophile, a general acid, or a general base based on the local environment surrounding the residue. During a reaction, a phosphate can be transferred to either small molecules or proteins from the histidine in the catalytic site, thus forming a phosphohistidine intermediate.

Kinases phosphorylate other substrate proteins, while phosphatases dephosphorylate the phosphoprotein substrates. The NME family of proteins are the only known His kinases in metazoan; however, not all the members of the 10-member NME family function the same. Here, we explore the structural and functional classification of the NME family of proteins and then examine the biochemistry of three reported pHis phosphatases: phosphohistidine phosphatase 1 (PHPT1), phospholysine phosphohistidine inorganic pyrophosphate phosphatase (LHPP), and phosphoglycerate mutase 5 (PGAM5). The recent literature has identified substrates of these His kinases and phosphatases, which may play a critical role in a wide range of cellular functions. An additional layer of regulation is added to the His kinases and phosphatases through other post-translational modifications (PTMs), such as oxidation and CoAylation, which affect their structure and function. Much like other phosphoamino acids, the complex interplay of the His kinases and phosphatases underpins their dichotomous roles as tumor suppressors and promoters, which is discussed further in the review. Finally, we discuss the development of antibodies, mass spectrometry methods, and small molecule modulators of His kinase and phosphatase activity used to study the pHis modification.

## 2. NMEs Are the Only Characterized His Protein Kinases in Mammals

### 2.1. NME Family Introduction

Initially investigated for their nucleoside diphosphate kinase (NDPK) activity [5], NME family protein members were also recognized as housekeeping genes [6]. NDPK enzymes catalyze the transfer of phosphate groups from nucleoside triphosphates (NTPs) to nucleoside diphosphates (NDPs) (EC number: 2.7.4.6) [5]. However, subsequent research uncovered an additional dual function as a histidine protein kinase [7]. This discovery expanded our understanding of the diverse roles these proteins play in cellular signaling and regulation.

All eukaryotic organisms have been found to possess His kinases, among which the most well-explored are NME1 and NME2 (also referred to as NM23 for non-metastatic protein 23). These kinases were named based on their non-metastatic function, which was derived from the first gene that was discovered to exhibit metastasis suppressor properties. Although the naming differs between organisms, these genes have been highly conserved through evolution. Examples are *awd* in the fruit fly *Drosophila melanogaster*, *ndk* in the roundworm *Caenorhabditis elegans*, *YNK1* in the yeast *Saccharomyces cerevisiae*, *gip17/guk* in the slime mold *Dictyostelium discoideum*, and *NmeGp1Sd* in the marine sponge *Suberites domuncula* [1]. While mammalian NME1 and NME2 are the best-studied enzymes in the 10-member NME family, there is increased interest in the roles the other 8 NME enzymes might play in various diseases.

**Figure 1:**
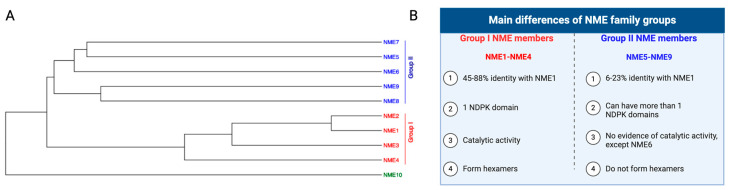
Comparison of NME family members: (**A**) Evolutionary tree of the sequence alignment of NME1–10 based on their amino acid sequences obtained from UniProt. (**B**) Main differences between Group I and Group II family members. Image created with BioRender.com.

The NME family is organized into two groups based on their sequence similarity and catalytic activity (Figure 1). Group I consists of NME1, NME2, NME3, and NME4, with the latter three sharing 45–88% overall identity with NME1 [8]. Group I members of the NME family have one catalytically active NDPK domain, and all have similar kinetic rates of catalysis [9]. Also, Group I members are capable of forming oligomers [10]. NME1/2 may exhibit different oligomerization states that could incline its phosphotransferase activity toward NDP phosphorylation or protein–histidine phosphorylation [11]. On the other hand, Group II consists of NME5, NME6, NME7, NME8, and NME9, which only share 6–23% overall identity with NME1 [8] (Figure 1). Some Group II members have more than one NDPK domain, although there is currently no evidence that these secondary domains are active. Additionally, it is unknown if members of this group are capable of forming oligomers. For example, a recent study found that NME6 was unable to form oligomers, and its NDPK domain only possessed activity when using recombinant protein [12], but another study reported that it was inactive [13]. Furthermore, no NDPK activity has also been observed for NME7 in two studies [13,14]. However, a study from our group indicates that NME7 undergoes autophosphorylation to generate 1-pHis NME7 in mouse, human, and bacteria cells, although data were not presented [15]. Contrary to Group I, which is expressed in many types of cells, most members of Group II are only expressed in ciliated cells, except for NME6 [12]. The final member, NME10, also known as XRP2, is separate from these two groups; it is still not well characterized and appears to have a different evolutionary path compared to the rest of the family [16].

There is ongoing discussion regarding functional redundancy among the Group I members of the NME family. Single knockout mice for NME1, NME2, and NME3 are viable [17,18,19,20], which may suggest partial compensation by other NME family members. In contrast, the NME1 and NME2 double mutant is embryonically lethal [21], underscoring the necessity of depleting both related NME members in studies to eliminate potential compensatory effects. NME1 and NME2 are closely related to each other, sharing 88% of their amino acid sequence, and are organized in tandem on chromosome 17q [22]. The other Group I NME family members, NME3/4, are localized on chromosome 16 [1]. Additionally, the Group II members, NME5–9, are located on chromosomes 5, 3, 1, 7, and 3, respectively, with NME10 situated on the X chromosome [1].

**Figure 2:**
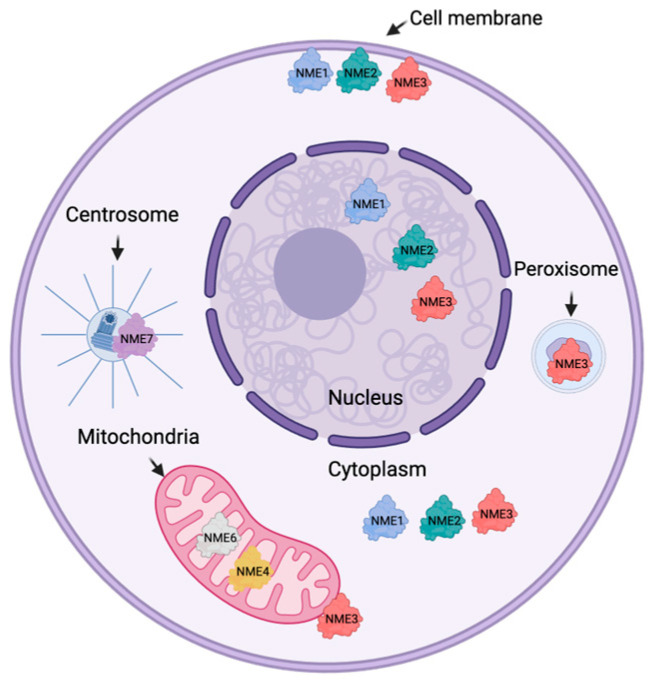
Known subcellular localization of NME family members in eukaryotic cells. Image created with Biorender.com.

The subcellular localization of members of the NME family has also become an area of interest in recent years (Figure 2). NME1, NME2, and NME3 are found localized to the plasma membrane, cytoplasm, and nucleus, while NME4 is found in the mitochondria [10,23]. NME3 has also been reported to be associated with the mitochondrial outer membrane [24] and peroxisomes [25]. Less is known regarding the localization of Group II members. NME6 is known to localize to the mitochondria [12], and there is evidence that NME7 can be found in the centrosome [14].

### 2.2. NME Enzymatic Activity

The primary function of the NME enzymes is their nucleoside diphosphate kinase (NDPK) activity, in which the γ-phosphate group is transferred from a nucleoside triphosphate, such as ATP, to a nucleoside diphosphate. However, the NMEs can also exhibit other enzymatic functions, including histidine protein kinase activity, serine–threonine protein phosphotransferase activity, geranyl and farnesyl pyrophosphate kinase activity, and possibly 3′-5′ exonuclease activity.

The serine–threonine protein phosphotransferase activity of NME in the presence of 1 M urea has been demonstrated using purified NME1/2 proteins from humans, fruit flies, yeast, and slime mold [26]. This function depends on the His118 catalytic site, which is also essential for its NDPK activity [26]. Its geranyl and farnesyl pyrophosphate activity has been reported with the phosphorylation of geranyl and farnesyl pyrophosphate by purified NME1 from rat and transfected human breast carcinoma cell lines extracts to give product to geranyl and farnesyl triphosphates [27]. This activity was inhibited when the NME1 His118 active site was mutated [27]. Finally, its 3′-5′ DNA exonuclease activity was observed using purified NME1 from *E. coli*; this exonuclease activity has been proposed to be crucial for NME1’s role in DNA repair [28]. Interestingly, this activity was independent of its His118 catalytic site and instead associated with the Lys12 residue, as evidenced by significantly lower exonuclease activity in the K12Q mutant form [28]. This finding supports the conclusion that the observed activity is due to NME1 itself and not a contaminant from the *E. coli* preparation, addressing concerns raised in this study [28].

NME histidine protein kinase activity has gained more attention recently with the development of monoclonal antibodies specific for 1- and 3-pHis [15]. Histidine kinase activity has been associated with tumor progression [29,30] and has been reported to play an important role in several types of cancer, such as neuroblastoma [29] and breast cancer [30].

### 2.3. Histone H4 Histidine Kinase

While NME1 and NME2 are currently the best characterized His kinases, there is another enzyme that has been reported to be capable of phosphorylating histidine residues in histone H4 and, therefore, has been termed as the Histone H4 histidine kinase (HHK). Histidine phosphorylation of histone H4 was first described in yeast [31] and has been reported in several other systems such as rat liver [32,33], rat carcinosarcoma cells [33], porcine thymus [34], human liver, and hepatocellular carcinoma cells [35], where HHK phosphorylates residues H18 and H75 [36]. While HHK phosphorylation typically appears to result in the formation of 1-pHis [32], in rat carcinosarcoma cells, phosphorylation was reported to result in the 3-pHis isomer [37]. Further studies on this enzyme are limited, and advancements in our understanding have been slow [38]. Currently, only a yeast HHK form has been purified, whereas no mammalian form has yet been characterized [31].

## 3. Histidine Phosphatases

### 3.1. PHPT1

Of all the known histidine phosphatases, PHPT1 (EC number: 3.9.1.3) is perhaps the most well-studied in the context of its histidine phosphatase activity. Also referred to as the 14 kDa phosphohistidine phosphatase, PHPT1 was independently identified by two research groups [39,40] in 2002 and is structurally distinct from other known protein phosphatases. Characterization of the enzyme revealed that it could hydrolyze a short synthetic pHis peptide and the bacterial protein CheA, which is known to contain pHis. PHPT1 was found to be resistant to vanadate and okadaic acid, inhibitors commonly used to block tyrosine and serine/threonine phosphatase activity, respectively, suggesting a mechanism of action different from these phosphatases. Early mutagenesis studies revealed several histidine and arginine residues, in particular His53 and His102, as being important for the catalytic activity of PHPT1 [41]. Soon afterward, the first crystal structure of PHPT1 was solved, which proposed His53 and surrounding residues as being the active site. This was based on the high conservation of these residues in PHPT1 orthologues across several animal species, a large positively charged surface surrounding H53, and docking predictions of short peptides based on known PHPT1 substrates [42]. NMR solution structures found that the addition of phosphate to the protein resulted in its binding to the His53 pocket region with a calculated K_d_ of 1.1 mM, which is similar to other phosphatases [43]. Based on data from phosphate and phosphate-free structures and an ^18^O labeling experiment, PHPT1 was proposed to have a catalytic mechanism in which His53 acts as a general base to activate a water molecule, leading to direct hydrolysis of the phosphate from the substrate without a phospho-enzyme intermediate. This mechanism is currently believed to be unique among the known protein histidine phosphatases.

In addition to pHis, there is limited evidence to suggest that PHPT1 may also act on phospholysine [44]. Using chemically phosphorylated 30 kDa and 90 kDa polylysine constructs, PHPT1 was found to be capable of completely dephosphorylating all phosphate groups from these molecules. Similar experiments using histone H1.2, which contains no histidine residues but does have lysine residues that can be chemically phosphorylated, showed that PHPT1 could dephosphorylate phospholysine with kinetic rates comparable to other pHis-based substrates.

### 3.2. LHPP

In contrast to PHPT1, LHPP (EC number: 3.6.1.1) is one of the least well-understood pHis phosphatases. LHPP (phospholysine phosphohistidine pyrophosphate phosphatase) was initially isolated from bovine liver as a 56 kDa protein comprised of a 33 kDa subunit dimer [45]. This phosphatase was later found to dephosphorylate free phospholysine, phosphohistidine, pyrophosphate, and imidophosphate [46], which led to its naming. The same research group subsequently cloned its human homolog and reported similar enzymatic activities as its bovine form [47]. LHPP belongs to the haloacid dehalogenase (HAD) family of phosphatases, characterized by the conserved catalytic core of two Asp residues separated by one amino acid, in which the first Asp functions as the nucleophile forming a phosphoaspartate intermediate [48]. In LHPP, this catalytic site is Asp17. In contrast to other HAD enzymes, LHPP is unique in that it has a Ser rather than an Asp in the +2 position after the catalytic Asp [48]. The ability of LHPP to dephosphorylate pHis-containing proteins is currently uncertain, as its crystal structure suggests that the catalytic site may not actually be accessible to proteins [48]. Furthermore, other members of the NagD clade of HAD family phosphatases, to which LHPP belongs, have been identified to only act on small molecules rather than proteins [48]. LHPP gained more prominence in 2018 when a study revealed its critical role in hepatocellular carcinoma and demonstrated an inverse correlation between LHPP expression levels and pHis levels [49]. Several studies have also highlighted the crucial role of LHPP in tumor progression in other cancer types [50]. However, LHPP’s molecular mechanism of action remains poorly understood, and further research is needed to identify its direct targets, whether they are proteins or small molecules.

### 3.3. PGAM5

PGAM5 (EC number 3.1.3.16) is a mitochondrial Ser/Thr phosphatase that has been recently described as also possessing pHis phosphatase activity [51]. Although it is primarily located in the mitochondria, its submitochondrial localization is uncertain as different studies have reported its presence in various parts of the organelle [52,53,54]. It has also been reported to be released into the cytosol following the loss of mitochondrial membrane potential [54]. PGAM5 belongs to the PGAM family of phosphatases characterized by having a conserved PGAM (phosphoglycerate mutase) domain responsible for phosphatase and mutase activity, although PGAM5 lacks the latter function [55]. PGAM5 exists in both a multimeric dodecamer and a dimeric form [56,57] with a conserved catalytic PGAM domain, which includes the His105 residue necessary for phosphatase activity [58]. Unlike PHPT1, PGAM5 forms a pHis intermediate during catalysis, a characteristic observed in many members of the PGAM family that function as mutases. Its C-terminal region contains the catalytic domain responsible for forming dimers, while a motif in the N-terminal is responsible for the assembly of dimers into a dodecamer form and is necessary for maximal phosphatase activity [56]. Utilizing cryogenic electron microscopy (cryoEM), a recent investigation has revealed the formation of dodecamers of PGAM5 in solution, which are assembled into filaments both in vitro and within cells [59]. Moreover, PGAM5 oligomerization was also found to modulate its activity [59]. Although many pSer/pThr phosphatase targets have been identified, NME2 remains the only pHis target identified for PGAM5 [51,55]. It is currently unknown whether PGAM5 has additional pHis targets, and identifying them will be crucial to understanding its role in various biological processes, including mitochondrial dynamics, cell death, and the immune response [55].

### 3.4. PP1/PP2A/PP2C

Better known for their pSer/pThr phosphatase activity, PP1, PP2A, and PP2C have also been shown to possess histidine phosphatase activity in vitro [60,61]. Traditionally, catalysis is achieved through two divalent metal ions binding within the active site and activation of a water molecule to hydrolyze the phosphate group from the enzyme–substrate [62]. While it is not known if hydrolysis of pHis is achieved through this mechanism, the evidence that exists seems to support this conclusion. For example, PP2C, which requires Mg^2+^ ions to function, did not hydrolyze pHis in the absence of Mg^2+^. Additionally, the use of a PP1 inhibitor blocked PP1-mediated pHis hydrolysis but did not impact PP2A or PP2C pHis phosphatase activity. Interestingly, PP2B does not hydrolyze pHis despite having a similar active site to that of PP1 and PP2A, possibly suggesting that there are some differences in the innate phosphoamino acid substrate specificity of these phosphatase family members.

## 4. Substrates of His Kinases and Phosphohistidine Phosphatases

Much like pSer, pThr, and pTyr, pHis has been found in many proteins and is responsible for a variety of different cellular processes. While the effects of a change in pHis state on protein function are largely unexplored, the evidence that does exist suggests that maintaining a careful balance between histidine kinase and histidine phosphatase activity is critical for proper cell function. This section focuses on highlighting many of the known substrates of the histidine kinases and phosphatases, how their pHis sites were identified, and a brief glimpse of possible biological functions controlled by histidine phosphorylation.

### 4.1. The Ion Channel Proteins KCa3.1 and TRPV5 Are Histidine Phosphorylated by NME2 and Are Dephosphorylated by PHPT1

**Figure 3:**
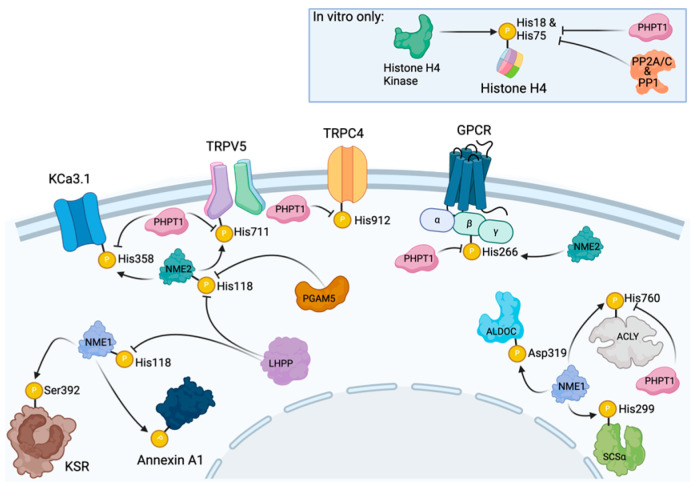
Histidine protein kinases and phosphatases in eukaryotic cells. NME2 and PHPT1 reversibly dephosphorylate His358 and His711 of the ion channel proteins KCa3.1 and TRPV5, respectively. TRPC4 is another ion channel protein histidine dephosphorylated by PHPT1 on His912. NME2 and PHPT1 phosphorylate and dephosphorylate His266 of the G beta subunit of G-protein coupled receptors (GPCR). ACLY is a cell metabolism protein reversibly phosphorylated on His760 by NME1 and PHPT1. Succinyl-CoA synthetase alpha (SCSα) and ALDOC are two other metabolic enzymes that are phosphorylated by NME1 on His299 and Asp319, respectively. NME1 can also phosphorylate the kinase suppressor of Ras (KSR) on Ser392 and Annexin A1. LHPP dephosphorylates NME1 and NME2 on His118, and PGAM5 dephosphorylates NME2 on His118. Image created with BioRender.com.

KCa3.1, a calcium-activated membrane-bound potassium ion channel tetrameric protein, was found to interact with NME2 via a yeast two-hybrid library screen [63]. Overexpression of NME2 led to activation of the ion channel, and in vitro phosphorylation assays showed that incubation of KCa3.1 with NME2 resulted in phosphorylation of the former by the latter (Figure 3). Mutational analysis revealed that His358 in the KCa3.1 cytoplasmic tail was the target residue of this activity. This process was found to be important for CD4 T cell activation, providing one of the first examples of the relevance of pHis in biological processes. A follow-up study to investigate the mechanism of action revealed that the four His358s coordinate a Cu^2+^ ion in the inactive channel tetramer, which prevents phosphorylation by NME2 [64]. When Ca^2+^ levels increase in response to TCR simulation, Ca^2+^ binds to the calmodulin domain, inducing a conformational change which releases the Cu^2+^ ion and allows for His358 phosphorylation and subsequent opening of the channel. Overexpression of PHPT1 in CHO cells resulted in a decrease in KCa3.1 channel activity, while a catalytically inactive mutant (H53A) version of PHPT1 did not show the same effect [65]. These results were attributed to the dephosphorylation of His358 in KCa3.1, whose phosphorylation had previously been shown. PHPT1 reverses this phosphorylation, which results in channel closing.

In 2013, Cai et al. investigated the TRPV5 ion channel to determine if histidine phosphorylation could control the activity of this channel in a similar manner to the KCa3.1 channel [66]. Using HEK293 cells in whole-cell patch experiments, they found that co-transfection of both NME2 and TRPV5 resulted in higher channel activity, which was not observed by co-transfection of catalytically inactive NME2 or transfection of TRPV5 alone. Based on previous studies, phosphorylation of His711 was predicted to be the residue in TRPV5 that was responsible for this observed effect. Transfection of wild-type TRPV5 or an H711N mutant (a nonphosphorylatable “mimetic” of His) in HEK293 cells and subsequent inside/out patch clamp tests showed that the H711N TRPV5 mutant exhibited reduced channel activation with NME2 than wild-type TRPV5 and that activation of the wild-type channel could be reversed by treatment with PHPT1.

The most recently discovered ion channel controlled by histidine phosphorylation is the TRPC4 ion channel. Although the histidine kinase involved in this process has yet to be identified, regulation of TRPC4 by PHPT1 was reported by Srivastava et al. [67] in a study investigating the potential role of PHPT1 in insulin signaling. Upon activation of TRPC4 by leptin or cesium, pancreatic β-cells from *Phpt1*^−/−^ mice were found to have reduced current flow when compared to β-cells from *Phpt1*^+/+^ mice, suggesting that PHPT1 is required for proper channel activation. Data from INS-1 cells transfected with GFP-TRPC4a showed comparable results with shRNA knockdown of PHPT1, causing reduced current levels. TRPC4 expression levels, as determined by GFP fluorescence, were similar between control and shRNA-treated cells, suggesting that the difference in current is not the result of changes in ion channel expression. Mutational analysis of histidine residues in the C-terminal region of TRPC4 revealed His912 as the likely target of phosphorylation as the H912N mutant was able to respond to leptin activation even upon shRNA knockdown of PHPT1. I/O patch clamp experiments with INS-1 further confirmed these results, as H53A PHPT1 was unable to activate the channel, and H912N TRPC4 showed higher activity compared to wild-type TRPC4 upon the addition of stimulatory levels of Ca^2+^.

### 4.2. The G Protein β Subunit Is Histidine Phosphorylated by NME2 and Dephosphorylated by PHPT1

In addition to interacting directly with surface receptors, NME2 can also phosphorylate the β subunit of the heterotrimeric G protein on protein on His266, an immediate downstream target of GPCR receptor proteins [68]. This phosphorylation occurs on a complex of NME2 with Gβγ dimers [68]. As it had been shown that the Gβ subunit could be histidine phosphorylated, Maurer et al. set out to determine if PHPT1 was the phosphatase responsible for dephosphorylation. Incubation of protein fractions containing Gβγ and NME2 with [γ-^32^P]GTP provided a strong signal on both NME2 and Gβ. Upon the addition of PHPT1, the signal on Gβ, but not NME2, was significantly reduced. Analysis of the wild type of PHPT1 overexpressed H10 and C3 cells showed a decrease in Gβ phosphorylation in the cells overexpressing PHPT1, despite Gβ protein levels showing no difference between the samples. Interestingly, NME2 was found to have both upregulated protein levels and increased enzyme activity in the PHPT1 overexpressed cells, which the authors attribute to a possible compensation mechanism to overcome the dramatic increase in PHPT1 protein [69].

### 4.3. The Cell Metabolism Proteins ACLY and SCSα Are Histidine Phosphorylated by NME1 and ACLY Is Dephosphorylated by PHPT1

Aside from its upstream cell signaling targets, NME1 is capable of phosphorylating ATP-citrate lyase (ACLY), an enzyme involved in fatty acid metabolism. This was discovered when the addition of purified and phosphorylated NME1 to PC12 rat pheochromocytoma cell cytosol resulted in the phosphorylation of a 120 kDa protein, which was later identified to be ACLY, with phosphorylation occurring on the N3 position on its catalytic site His760 [70]. In a similar manner, NME1 was first found to form a complex with SCSα, and it was suggested that this interaction activated SCSα by phosphorylation in rabbit mitochondrial preparations [71]. This phosphotransfer activity from NME1 to SCSα was then demonstrated through phosphorylation of SCSα when incubated with recombinant NME1 and NME2 wildtype forms but not with NME1 catalytic mutant form (H118F) [72]. They also showed a decrease in phosphorylated SCSα with a “killer of prune”-mutant NME1 (P96S) [72,73]. The interaction between NME isoforms and SCSα has also been reported in the mitochondria of pancreatic β cells [74]. It is suggested that the active site His299 of SCSα is phosphorylated by NME1 [75,76].

As with other NME substrates, PHPT1 can also dephosphorylate ACLY. Klumpp et al. [77] utilized a previously reported procedure in which [γ-^32^P]ATP is added to the sample in the presence of EDTA to enrich for phosphorylation of histidine residues. This method was applied to rabbit liver, which resulted in the appearance of three bands at 110 kDa, 40 kDa, and 20 kDa under denaturing conditions. The addition of PHPT1 to these samples revealed that the 100 kDa band, but not the 40 kDa and 20 kDa bands, showed reduced intensity, which was dependent upon both the amount of PHPT1 used and the duration of incubation. Further purification and analysis revealed this protein to be ACLY. In an in vitro specificity experiment with other phosphatases, it was discovered that alkaline phosphatase and PP2A were also capable of dephosphorylating ALCY, raising the possibility that a single pHis site may have multiple layers of regulation controlling it.

### 4.4. Histone H4 Is Phosphorylated by HHK and Dephosphorylated by Several Phosphatases

As mentioned previously, histone H4 can be phosphorylated by an unidentified protein kinase referred to as histone H4 histidine kinase (HHK) [32,34,35,36,37,38]. Interestingly, several studies have reported that histone H4 can be reversibly dephosphorylated by PHPT1. Although it has not been demonstrated in vivo, histone H4 has been reported as a PHPT1 substrate through in vitro testing. An initial report from Atwood et al. [78] found that peptides corresponding to H18 and H75 of H4, which had been chemically phosphorylated (and thus contained a mixture of 1- and 3-pHis species) could both be dephosphorylated by PHPT1. Kinetic data from the experiment revealed that PHPT1 appears to have a slight preference for 1-pHis over 3-pHis, but *k*_cat_/*K*_M_ values for both substrates were within two-fold of each other. However, in this same publication, PHPT1 was reported as being unable to dephosphorylate histone H4 when phosphorylated with the histone H4 kinase. A later report by Beckman-Sundh et al. [79] found that H4, which had been phosphorylated using phosphoramidate, was able to be dephosphorylated by PHPT1.

In addition to PHPT1, the Ser/Thr phosphatases PP1, PP2A, and PP2C have also been shown to possess histidine phosphatase activity towards H4 [60]. Using HHK and [γ-^32^P]-ATP, H4 was radiolabeled on the H75 target histidine residue. All three phosphatases were able to dephosphorylate H4, with PP2A and PP2C doing so in a dose-dependent manner. A PP1 inhibitor or okadaic acid (PP1/PP2A inhibitor) caused a decrease in phosphatase activity toward H4 that was not observed with PP2C, which is unaffected by these compounds. Kinetic analysis of the histidine phosphatase activity revealed that all three enzymes have *k*_cat_/*K*_M_ values for H4 that are as good as or better than a known substrate of each (phosphorylase a for PP1/2A and myosin P-light chain for PP2C) [61].

### 4.5. ALDOC and KSR Are Phosphorylated by NME1 but Not in a Histidine Residue

Aldolase C (ALDOC) is another metabolic enzyme that has been reported to be phosphorylated by NME1. The first hint that NME1 phosphorylates aldolase C was from experiments with recombinant NME1, where a 43 kDa protein present in bovine brain extracts was found to be phosphorylated on an aspartate residue [73]. A follow-up study determined that the 43 kDa protein was aldolase C and that the phosphate was being transferred from NME1 to the Asp319 residue of aldolase C [80]. However, it is still unknown if PHPT1 or another phosphatase catalyzes the dephosphorylation at this residue. Interestingly, the sequence surrounding aldolase C D319 residue is very similar to that of the histidine phosphorylated residues in ACLY and SCSα.

Kinase suppressor of Ras (KSR), a scaffolding pseudokinase protein that plays a crucial role in the ERK MAPK signaling pathway, has also been reported to be phosphorylated by NME1 on a non-histidine residue [81]. In a study using transfected HEK293T cells and MDA-MB-435 breast carcinoma cells, HPLC of phosphorylated KSR tryptic peptides and site-directed mutagenesis revealed that Ser392 is phosphorylated by NME1 [81]. However, as with aldolase C, no phosphatase that targets this phosphosite has been identified.

Another NME1 substrate that still lacks an identified phosphatase counterpart is annexin A1, which was reported to be histidine phosphorylated by identifying a 37 kDa purified phosphoprotein from ovine tracheal epithelia that was phosphorylated by NME1 and 2 but was not affected by inhibition of Ser/Thr and Tyr kinases [82]. By performing phosphoamino acid analysis, it was determined that annexin A1 is histidine phosphorylated; however, the specific site of phosphorylation remains unknown [82].

### 4.6. PGAM5 Can Directly Dephosphorylate His105 on NME2

Despite being one of the more recently identified histidine phosphatases, at least one target of PGAM5 histidine phosphatase activity has been discovered. In a report by Panda et al. [82], overexpression of PGAM5 and NME2 in HEK293 cells and subsequent pulldown of PGAM5 revealed NME2 as a binding partner. A catalytically inactive H105A PGAM5 mutant could also bind NME2, but experiments with overexpression of NME1 or NME3 did not result in detection of these proteins upon PGAM5 pulldown, suggesting this interaction is likely specific and not dependent upon the enzymatic activity of PGAM5. An in vitro assay found that incubation of PGAM5 with NME2 resulted in a time-dependent decrease in NME2 1-pHis levels, while PGAM5 H105A had no effect. Immunoblotting of HEK293 cells with co-overexpressed NME2 and PGAM5 showed similar results. Residues 77–88 of PGAM5 were found to be critical for NME2 binding, as a construct lacking this region failed to dephosphorylate NME2. As discussed above, NME2 is required for activation of the KCa3.1 ion channel through phosphorylation of its H358 residue. Overexpression of PGAM5 was found to reduce channel activity, while shRNA knockdown resulted in increased channel activity. Consistent with the above data, the expression of PGAM5 H105A did not have any effect on channel activation. Further in vitro experiments ruled out PGAM5 as directly dephosphorylating KCa3.1, suggesting that the observed changes in channel activity are a result of its activity towards NME2.

### 4.7. LHPP Dephosphorylates His118 NME1/2

The identification of direct substrates that are dephosphorylated by LHPP has not been well studied. One study shows the critical role of LHPP in stress-related depression in a mouse model that identified both NME1 and NME2 as substrates for LHPP. It was found that the 1-pHis signal in cell lysates of HEK293T cells transfected with NME1-Flag and NME2-Flag lysates decreased when co-transfected with myc-LHPP [83]. Additionally, purified 1-phosphohistidine GST-NME1 and GST-NME2 were dephosphorylated when incubated with purified GST-LHPP in a time-dependent manner. Another study reported that LHPP induced the ubiquitin-mediated degradation of PKM2 in glioblastoma; however, phosphorylated PKM2 was not a direct target of LHPP [84].

To address the absence of known direct targets for LHPP, our team recently utilized proximity-labeling proteomics to identify potential binding partners by stably expressing the LHPP-TurboID fusion protein in MDA-MB-231 and MCF7 breast cancer cell lines [85]. The results of gene ontology gene set enrichment analysis indicated that in MDA-MB-231 cells, LHPP interactors are involved in biological processes such as histone modification, RNA processing, and actin filament organization. The study identified key proteins such as PAK2 and ROCK2 as LHPP interacting partners. These proteins play pivotal roles in orchestrating cytoskeletal organization and are integral components of the TGF-β signaling pathway, the latter of which LHPP has been previously implicated [86]. More studies on the direct targets of LHPP are needed for a better understanding of its molecular mechanism.

## 5. PTM and Stress Regulation of NME Catalytic Activity

Mammalian His kinases NME1/2 and phosphohistidine phosphatase PHPT1 have been studied for their multifunctional roles in cells. However, the regulation of their enzymatic activities is poorly understood. In this section, we will discuss possible mechanisms and PTMs that may play a role in controlling these activities.

### 5.1. H_2_O_2_- or Diamide-Triggered Oxidation

The regulation of NME1/2 activity by oxidative stress has been studied for over two decades. Under oxidative stress, NME1/2 undergoes disulfide cross-linking when treated with diamide (a disulfide-stress inducer) or H_2_O_2_ in RIF-1 cells, which leads to the loss of catalytic NDPK activity [87]. Similar results were observed when recombinant NME protein was treated with H_2_O_2_, where decreased NME1/2 enzymatic activity upon oxidation correlates with the concentration of oxidation agent as well as incubation time. Under certain circumstances, this oxidative crosslinking can be reversible. For example, cells are able to self-recover under mild stress (0.5 mM diamide) but not under strong stress conditions (>0.75 mM diamide), and use of DTT, a reducing reagent, was able to restore the NDPK activity in recombinant protein [87]. In another study, glutathionylation, another form of oxidation, was also found to affect NDPK activity. After treating recombinant NME1 with glutathione, the NDPK activity was disrupted. The thioredoxin reductase 1 (TrxR1)-thioredoxin (Trx)-NADPH system can identify oxidized NME1 as a substrate and reverse the disulfide modification [88]. These results suggest that oxidation can be used as a mechanism for regulating NME NDPK activity in cells.

### 5.2. Non-Covalent CoA-Binding and Covalent CoAlation Regulate NME1/2

Very recently, several groups have discovered that NME1/2 are primary binders of CoA, long-chain fatty acyl-CoA (LCFA-CoA), and acetyl-coenzyme A in various cells and tissues [89,90,91]. CoA was also found to bind to other members of the NME group I family (NME3 and NME4) [89]. In vitro experiments showed that both CoA and LCFA-CoA competed with NME NDPK nucleotide substrates for binding to the nucleotide-binding site [89,91].

Under oxidative treatment with diamide, NME1 was found to be CoAlated on cysteine 109. In vitro experiments demonstrated that the CoAlation inhibited NME1’s NDPK activity, which was reversed by the addition of a reducing agent [89].

### 5.3. Structure Assembly Changes in NME1/2 upon Mutations, Oxidation, CoAlytion, and CoA-Binding

#### 5.3.1. Change in Oligomerization States

The oligomerization states of NME1/2 can impact their functional activities. Native NME1/2 forms hexamers that are essential for maximal NDPK activity. Regarding the protein kinase activity of NME1/2, it remains a mystery how the compact NME1/2 catalytic pocket can adapt to recognize protein substrates or whether changes in oligomerization might play a role.

Several mutations and different stresses can impact oligomerization, disrupting the interconversion of hexamer to dimer or other intermediate states. Most of these changes reduce NME’s NDPK activity to varying degrees. However, not all mutations affect NME’s other functions in the same way.

In *Dictyostemlium*, the double mutation P100S and N150 stop results in NME1 forming dimers, which lack enzymatic activity. Interestingly, this form of NME1 binds to DNA, whereas the wild-type NME1 does not [11] (Table 1).

The S120G mutation in NME1 has been found in several aggressive pediatric neuroblastomas. In a purified protein system, NME1-S120G shows increased dimer and reduced hexamer formation. Functionally, it retains significant NDPK activity but was found to have decreased histidine autophosphorylation and histidine protein kinase activity when using SCSα as substrate. At the same time, overexpression of S120G did not suppress motility in the MDA-MB-435 melanoma cell line, a known response to increased wild-type NME levels [72]. S120 is located near the active site and forms a hydrogen bond with the side chain of E129, which also forms a hydrogen bond with H118-N3. Loss of the hydrogen bond between S120 and E129 might disrupt the stability of the catalytic pocket. S120G typically adopts a heteromeric state when crystallized, but it is not known if this is an artifact of crystal packing. An in-solution protein characterization method may provide insights into the protein in its native state, such as small-angle X-ray scattering (SAXS) and HDX-MS. Comparing the crystal structures of NME1 WT and NME1 S120G (PDB: 2HVD and 2HVE, both complexed with ADP), the two structures are highly similar overall, with no significant differences found in the active site. However, in in vitro biochemical experiments, purified NME1 S120G had decreased enzyme stability compared to the wild type as evaluated by urea- or temperature-induced denaturation experiments [92]. The NME S120G protein had a folding defect and accumulated more folding intermediate molten globule states [93]. The folding defect may be the reason for the more dimeric form of S120G. Further information about the whole protein’s flexibility and stability will be useful in drawing any conclusion about the reason for dissociation. To rescue the NDPK activity, TMAO (trimethylamine-N-oxide) was found to have a function of improving the folding and stimulating association of S120G, which then rescued the enzymatic activity of S120G [93]. However, this effectivity has not been tested in a cell model or mouse model. By the dissociation and conformational change, S120G might be able to change its protein binding partners. More information about the protein–protein interaction should be studied in the future.

**Figure 4:**
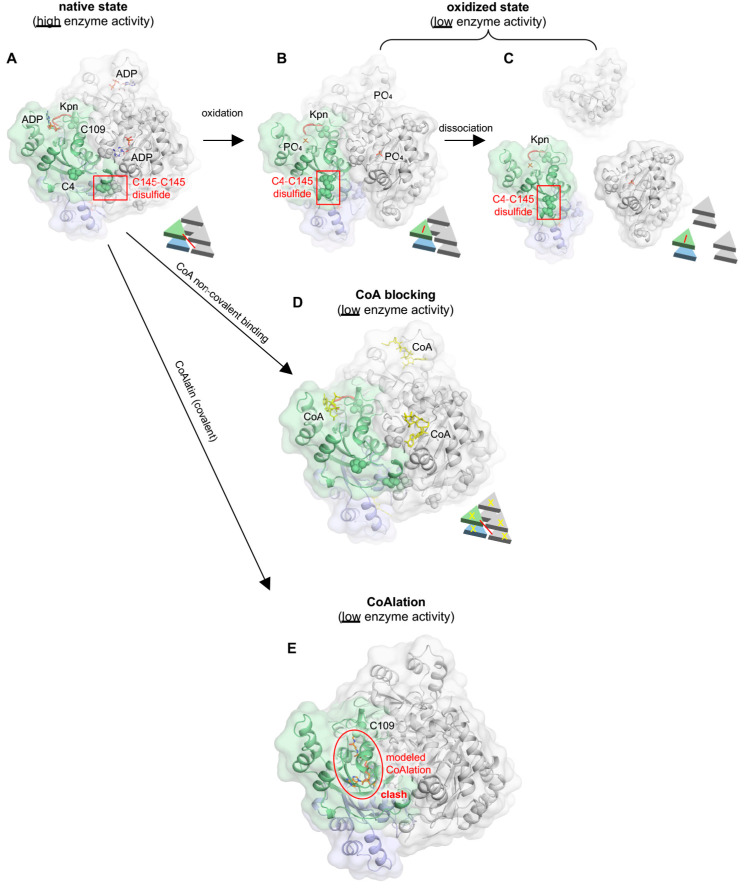
Effects of PTMs and natural inhibitors on oligomerization, structure, and enzyme activity of NME1. NME1 hexamers are shown in each panel. For clarity, only two monomers are shown in colors (green and blue), and the rest are all in grey. Cysteines are represented by spheres, while the Kpn loop (residues 109–114) in chains A (the green chain) is in red. A 6-triangle cartoon model is shown at the bottom-right corner of each panel to show the topology of the oligomers, where red lines represent inter- or intra-molecular disulfides, and yellow crosses represent non-covalent CoA inhibitors: (**A**) Native state (PDB 2HVD). (**B**) Oxidized state (PDB 4ENO). (**C**) A model of dissociated NME1 adapted from panel B (PDB 4ENO). (**D**) CoA-bound NME1 (PDB 7ZTK). (**E**) A covalent CoAylation is modeled onto C109 (PDB 2HVD).

A P97S (P96S in humans) mutation in *Drosophila* NME (Awd) has been shown to affect developmental phenotypes [94]. The loop that contains P96 was called Kpn (Killer of Prune) loop and was later found to be important to the integrity of the structure (Figure 4). Mutating this proline residue to serine has been shown to alter the oligomerization state [72,95] (Figure 4A). Another possibility is that S96 could form a hydrogen bond with C109 as they are close enough (approximately 2.5 Å) in the structure. P96S may affect the conformation of the Kpn loop and break the inter-subunit interface, thereby affecting the stability of the NME. Note that this dimer form of Awd does not significantly alter NDPK activity, which indicates that the dramatic phenotype change in *Drosophila* is due to other functions of Awd.

Another case of changing the oligomerization form of NME1 is via forming a complex with a protein partner, GAPDH. In vitro, purified NME1 has been shown to form a complex with GAPDH. The formation of this complex activates the protein phospho-transferase function of NME1 when using a heat-denatured CX-1 protein as the substrate. Interestingly, the complex between NME1 and GAPDH consists of dimers of NME1 bound with dimers of GAPDH and does not affect the NDPK activity of NME1 or the GAPDH activity compared to each of their native states (NME1: hexamer, GAPDH: tetramer) [96].

Upon H_2_O_2_ oxidation treatment in vitro, hexametric NME1/2 dissociated into dimers as well as other multimers between dimer and hexamer. C109, which is located at the interface between two monomers of NME1/2 on the Kpn loop, is important for hexamer formation [46,97]. Structural and mass-spectrometry experiments show that C109 does not form a disulfide bond under non-oxidative conditions [98]. However, under oxidative stress, it forms an intersubunit C109-C109 disulfide bond, which might distort the interface interactions, leading to dissociation of the hexamer and subsequent loss of NDPK activity. In line with this, C109A was found to be resistant to oxidative stress [7,88]. Compared to wild-type NME, the C109A mutant did not crosslink under oxidation and maintained similar NDPK activity to the wild-type enzyme [87]. NME1 has also been reported to be glutathionylated at C109 [88]. In another study, a comparison of the crystal structures of NME1 in its native and oxidized forms revealed that oxidation breaks the intermolecular C145-C145 (Figure 4) disulfide bond and forms an intramolecular C4-C145 disulfide, which might cause a conformational change and break the stable hexametric form (Figure 4) [98].

As discussed above, CoA has been shown to bind non-covalently to the active site of NME, and the co-crystal structure shows a stable hexamer of NME1 [89]. Under oxidative stress, C109 was found to be CoAlated, resulting in the loss of its NDPK activity. It is known that under oxidative stress, NME hexamers may break into dimers, but with CoAlation, no data were presented to show whether CoAlation can occur only in dimers or can happen in the hexamer state and prevent NME from being oxidized. Presumably, CoAlation will dramatically change the conformation of NME as the identified CoAlated site, C109, is buried under normal conditions [99].

A monomeric or dimeric form of NME is likely to be more flexible compared to the hexamer form, and thus, the protein may have more space to accommodate a protein substrate. Few monomeric species have been reported from in vitro purification. Presumably, a free monomer may not be very stable where its hydrophobic dimer interface surface is exposed, and thus the monomers would likely form homo- or hetero-oligomers to yield a more stable complex. It might also be a case that other proteins can bind to this hydrophobic region and allow NME1 to bind substrates that do not normally interact with the NME hexamer.

**Table 1:** Summary of mutations, corresponding oligomerization states with enzyme activity of NDPKs from different species.

Mutations /Conditions	Oligomerization State	NDPK Activity	Protein Kinase Activity	Other Function	Other Characterizations
*Dictyostemlium* P100S with N150stop [11]	Dimer	Dimer does not have NDPK activity	No assay performed	Can bind to DNA, while WT cannot	No assay performed
Human S120G [72]	Increased dimer	Not changed much to WT	Slightly decreased	Decrease Autophosphorylation signal	Found in several aggressive neuroblastomas. Not suppress cancer cell line mobility.
Human P96S [94]	On k-pn loop, increased dimer	Not changed compared to WT	Decreased	Similar to WT autophosphorylation signal	The killer of prune mutation of *Drosophila* Awd. Not suppress cancer cell line mobility.
Human Co-expression with GAPDH [96]	NME dimer complexed with GAPDH dimer	Not changed	No assay performed	GAPDH also maintained its activity	Only this complex, rather than the NME1 or GAPDH alone, has phosphotransferase activity.
Human Oxidation [87]	Dimer	No NDPK activity	No assay performed	No assay performed	C109A prevents loss of NDPK activity under oxidation.
Human CoAlation under oxidation stress [90]	No assay performed	No NDPK activity	No assay performed	No assay performed	C109-CoAlation
Human CoA compete for binding [89]	Not disrupt	Decreased along with concentration of CoA	No assay performed	No assay performed	No assay performed

#### 5.3.2. CoA Binds to the Active Site of NME (Non-Covalent)

Coenzyme A (CoA, 3′-phosphoadenosine diphosphate-pantetheine-β-mercaptoethylamine) or myristoyl-CoA can occupy the active site of NME1/2 (non-covalent) where the NDP or protein substrates would bind. The structure of the CoA-NME1 bound form shows a unique binding mode compared to the GDP- or ADP-bound form, as the α- and β-phosphates are oriented in a novel position within the catalytic pocket [99]. The binding of myristoyl-CoA with NME2 is very similar [91]. Furthermore, in both structures, NME-R58 is very important as it interacts with CoA’s phosphate group, which does not occur in the ADP/GDP-bound forms. NME1/2-R58E does not impair NDPK activity but is also incapable of binding CoA and its derivatives and is thus resistant to CoA-mediated NDPK activity inhibition.

### 5.4. Possible Physiological/Functional Changes Caused by Oxidation and CoAlytion

NME is primarily located in the cytosol, where the concentration of AcCoA is approximately 1 mM, comparable to the levels of NDPs, the substrates of NME. Under stress conditions, ATP levels might dramatically decrease, leading to increased competition between ATP and CoA/derivatives. In short, the inhibitory effect of CoA and CoA derivatives are physiologically relevant. Several studies below have investigated the physiological relevance of CoA/CoA derivative regulation of NME.

Increased LCFA-CoA in the MDA-MB-231 breast cancer cell model inhibited NME1/2’s NDPK activity and resulted in endocytosis inhibition. The R58E mutant exhibited the same level of NDPK activity compared to the wild type. Interestingly, NME1-R58E was unable to bind LCFA-CoA, was insensitive to oleic acid treatment, and showed no changes in endocytosis [91]. Furthermore, NME1 was found to have a tumor-suppressive role in mice with either WT or R58E NME1. In contrast, in high-fat diet (HFD) fed mice, wild-type NME1 lost the ability to act as a metastasis suppressor with MDA-MB-231 xenograft tumors, while NME1 R58E retained the ability. All these results indicate that LCFA-CoA is able to regulate NME1 function in vivo [91].

A significant accumulation of fatty acids was found in *Nme2*^−/−^ mice, suggesting that NME2 may help regulate fatty acid levels within cells. When these mice were exposed to HFD, they showed strong liver steatosis compared to the WT mice, along with high levels of triglyceride in the liver, suggesting that NME2 may play a role in the suppression of liver lipogenesis [90].

### 5.5. PHPT1

Regarding PHPT1, an early report from Martin et al. found that extensive oxidation of Met95 occurred along with modest oxidation of Met64, Cys69, and Cys71 upon the addition of 1 mM H_2_O_2_ [100]. Although modification of Met95 was expected to potentially cause a significant alteration in PHPT1 activity due to its proximity to the active site pocket, a mass spectrometry-based enzyme activity assay utilizing a small peptide substrate found that selective oxidation of Met95 did not affect enzyme activity. Further kinetic analysis revealed that oxidation of Met95 resulted in a 1.4-fold decrease in Km but no change to the kcat value when using pNPP as a substrate. However, a report several years later focused on the development of a new assay to detect PHPT1 activity in vitro found that the addition of reducing agents (β-mercaptoethanol, DTT, TCEP) could significantly increase PHPT1 activity up to 600% compared to conditions without reducing agents, which may suggest that PHPT1 is indeed sensitive to oxidation and that this oxidation may occur naturally under in vitro conditions [101]. To date, the importance of PHPT1 oxidation in a physiological context has not been investigated and thus remains an open question.

Proteomics studies have shown that a number of PHPT1 residues can be phosphorylated—the most prominent being Tyr93 and Tyr116. As phosphorylation is a frequently used PTM to control protein activity, uncovering the roles of these two residues may reveal a mechanism to control PHPT1 activity. Although information on PHPT1 phosphorylation remains scant, a doctoral thesis by Wang [102] provides the first glimpse into the possible function of tyrosine phosphorylation on this enzyme. Based on a report that Y93 could be phosphorylated upon the addition of erythropoietin to UT7epo cells [103], the JAK2 and Fyn tyrosine kinases were identified as possible candidate enzymes. An in vitro phosphorylation assay revealed that after 2 h, Fyn but not JAK2 was able to phosphorylate PHPT1. Activity assays revealed that after 2 h of incubation, PHPT1 activity decreased by 11% and 18% when using 500 μM and 1 mM ATP, while an overnight incubation with 1 mM ATP reduced activity by 34%. Mass spec analysis of the resulting protein revealed phosphorylation on 4 different tyrosine residues: 52, 91, 93, and 97. An in vitro phosphatase activity assay using PHPT1 Tyr to Phe mutants found that after an overnight incubation with Fyn, only the Y93F mutant showed no decrease in enzyme activity, suggesting that phosphorylation of this residue might be involved in negative regulation of PHPT1 activity.

A study by Sakai et al. [69] revealed that PHIP-1, the *C. elegans* ortholog of PHPT1, could interact with the Ser/Thr kinase UNC-51 (ULK1 ortholog). By performing an alanine scan screen of Ser/Thr residues in the protein, Ser112 was found to be the target site of UNC-51 kinase activity. Phosphorylation of PHIP-1 Ser112 resulted in increased histidine phosphatase activity. An in vitro PHIP-1 activity screen utilizing pHis-containing GPB-1 (G protein β ortholog) and CheA (a bacterial pHis protein that can act as PHPT1 substrate) showed that the S112A mutant possessed little phosphatase activity, while a phosphomimetic mutant S112E was able to efficiently dephosphorylate substrates. The authors note that the equivalent residue in human PHPT1 would be Thr119 and suggest that PHPT1 activity might be controlled through its phosphorylation, but proteomics experiments have yet to identify the phosphorylation of this residue.

## 6. Signal Regulation of Histidine Phosphorylation in Cells by NME and pHis Phosphatases

### 6.1. NME1/2 and Phosphatases in Cancer

An increasing number of studies implicate NME1 and NME2 in cancer, but depending on the cancer context, they can either be protumorigenic or tumor suppressive. On the one hand, NME1 expression was originally identified as a suppressor of metastasis in melanoma and epithelial cancers [104,105,106,107,108,109]. On the other hand, NME1 expression correlates with aggressive neuroblastoma tumor features [110]. The apparently contradictory role of NME1 and NME2 could be explained by differences in the cellular context. Most of the published studies have focused on the role of NME1 and NME2 in cancer independently of their histidine kinase function, which might help explain some of the contradictory results observed thus far.

#### 6.1.1. Histidine Kinase NME1/2

##### NME1/2 as a Metastasis Suppressor

Most of the studies demonstrate that high NME1 RNA and protein levels are correlated with a good prognosis in cancer, including breast cancer, ovarian cancer, cervical cancer, head and neck carcinoma, and melanoma. Furthermore, overexpression of NME1 in these cell lines reduced their metastatic potential [111].

NME1 inhibits the proliferation of tumor cells by regulating the MAP kinase pathway. Mechanistically, NME1, through its histidine kinase function, phosphorylates KSR on S392 and activates KSR (kinase suppressor of Ras), leading to altered subcellular localization [81]. KSR promotes assembly of the Raf/MEK/ERK complex and activation of the ERK/MAPK signaling pathway, modulating cell proliferation [112,113,114]. NME1 overexpression in breast carcinoma cells can promote KSR phosphorylation and decrease MAPK activation. When NME1 is depleted in hepatocellular carcinoma (HCC), MAPK activity is increased through hyperactivation of ERK [115]. These results all suggest that NME1 regulates Ras/MAPK signaling through its protein kinase function. A recent paper demonstrated that ASH1L (histone-lysine N-methyltransferase enzyme encoded by the ASH1L gene) and its antisense lncRNA ASH1L-AS1 are highly expressed in gastric cancer, and they are associated with a poor prognosis. ASH1L-AS1 interacts with NME1 protein, modulating the RAS signaling pathway [116].

NME1 could suppress proliferation through other mechanisms. NME1 is poorly expressed in glioma tissue at the protein and RNA levels. Overexpression of NME1 negatively regulates the protein kinase C (PKC) signaling pathway and inhibits cell proliferation in glioma [117]. In nasopharyngeal carcinoma, miR-141-3p promotes cell proliferation by inhibiting NME1 expression [118].

Epithelial–mesenchymal transition (EMT) is a process by which epithelial cells are reprogrammed into a mesenchymal phenotype, usually the first step of metastasis. The role of NME1 in EMT has not been extensively studied, but one study demonstrated that depletion of NME1 promotes EMT in breast cancer (BCa), showing that NME1 is an inhibitor of EMT [119]. NME1 expression is negatively associated with EMT markers and is an inhibitor of pathways involved in EMT, such as AKT and MAPK. NME1 is also a negative regulator of TGF-β1, which promotes EMT in lung cancer. NME1 inhibition induces an increase in TGF-β1, suppressing epithelial marker E-cadherin and upregulation of the mesenchymal markers β-catenin and fibronectin [120].

EMT decreases cell adhesion and promotes migration and invasion, which are involved in the metastasis process. First, NME1 is a negative regulator of migration in different cancers such as oral squamous cell carcinoma, liver, glioma, breast, and colon cancer. It has been demonstrated that NME1 could be localized at lamellipodia, which are important structures for cell migration [121]. NME1 is known to modulate the Rho-Rac pathway, which is involved in cell migration. In different cancer cell types, NME1 could bind to gelsolin, thereby inhibiting the actin depolymerizing function of gelsolin and decreasing cell migration [122]. NME1 negatively regulates TIAM1 and inhibits Rac activation, which is involved in cell migration [122]. NME1 also interacts with Db1-1, which is a specific Rho GTPase CDC42 exchange factor. The association of NME1 and Db1-1 causes inactivation of CDC42 and thereby inhibits migration and tumor progression [123]. In colorectal carcinoma (CRC), NME1 inhibits the myosin light chain (MLC), resulting in reduced cell migration [124]. Furthermore, Boissan et al. elegantly demonstrated that depletion of NME1 promotes migration of the cells and reorganization of the actin cytoskeleton [115]. A new study demonstrated that NME1 modulates RKIP (Raf kinase inhibitory protein) expression, promoting BCa cell migration. NME1 has a low expression in BCa, but when the BCa cells are treated with epirubicin (a DNA-damaging drug used for BCa treatment), there is an increase in NME1 expression, promoting an overexpression of RKIP and inhibition of the migration of the BCa cells [125].

Other mechanisms implicate NME1 in migration, such as integrin regulation or downregulation of receptors. In melanoma, NME1 promotes focal adhesion through the integrin β3 (ITGβ3) protein. Mechanistically, NME1 induced ITGβ3 gene transcription and upregulated ITGβ3 protein levels on the cell membrane, which is expected to decrease cell migration through the stabilization of focal adhesions [126]. In HCC, NME1 is able to regulate glycosylation of integrin β1 and down-regulate integrin β1 subunit on the cell surface, inhibiting migration [127]. Furthermore, NME1 could inhibit migration by downregulation of the lysophosphatidic acid receptor EDG2 [128]. In BCa, NME1 loss could be due to the downregulation of CTCF and EGR1, promoting an increase in cell migration [129].

Little is known about the role of NME1 as a protein kinase or histidine kinase in migration. Another important mechanism by which NME1 modulates migration is through its interaction with dynamin (an atypical large GTPase) which plays a key role in endocytic vesicle scission. Mechanistically, NME1, through its NDPK function, generates the GTP needed for dynamin function, decreasing BCa cell migration through the internalization of chemotactic receptors in migrating cells [130,131]. Regarding the histidine kinase function of NME1, it has been shown that NME1 inhibition of cell migration could be mediated through its protein kinase function. In order to understand the biological effects related to NME’s function as a protein–histidine kinase, site-directed mutagenesis of the catalytic site has been performed through mutation of H118 into phenylalanine to prevent its autophosphorylation, which is essential for NME kinase function [132]. Since H118 is important for both nucleotide and protein–histidine kinase function, to distinguish between these two functions, the separation of function of P96S has been used, which prevents protein but not NDP phosphorylation. This mutation works by causing conformational changes in NME1, disrupting its binding to its protein substrate [30]. Overexpression of NME1 in BCa increases 1-pHis levels and decreases migration. Surprisingly, migrating cells overexpressing NME1 were low in their levels of 1-pHis compared to stationary cells. These data suggest that NME 1-phosphohistidine levels are controlled during tumor cell migration and that there is no correlation between total NME levels and its 1-phosphohistidine form in cells exhibiting active motility.

When NME1 P96S is expressed in BCa cells, NME1 1-pHis levels are higher than those in the WT protein, as detected by immunoblotting, and higher NDPK activity was detected. Neither the NME1 P96S nor the NME1 H118F mutation affected BCa cell migration. These data suggest a suppressive function of histidine kinase activity of NME1 in cell migration [30].

In order to metastasize, cells need to invade and degrade the extracellular matrix (ECM). NME1 decreases the invasion of cancer cells in BCa, melanoma, hepatoma, HCC, colon carcinoma (CRC), and neuroblastoma. NME1 expression could be negatively regulated by miR-146a or miR-139-5p in BCa and HCC, respectively, promoting the invasion of the cells [133]. In non-invasive human HCC and CRC cell lines, NME1 depletion increases their metastatic properties. Here, NME1 inhibits extracellular matrix invasion. Mechanistically, NME1 decreases the formation of invasion structures (invadopodia) through downregulation of the secretion of matrix metalloproteinases (MMPs) required for invadopodia-mediated degradation of ECM [115]. Another study demonstrated that loss of NME1 increases MT1-MMP at the cell surface, promoting ECM degradation and the invasive potential of BCa cells [134]. HSP90AA1 interacts with NME1 and increases its lifetime by impeding its ubiquitin-proteasome-mediated degradation, reducing the metastatic potential of BCa cells [135]. In melanoma, NME1 interacts with the p110 subunit of PI3K, inhibiting p110 function and phosphorylation of AKT. When NME1 is depleted, p110 and AKT are activated, promoting migration and invasion of the cancer cells [136].

Whereas the role of NME1 in cancer has been extensively studied, the role of NME2 is less well understood [23]. However, a metastasis suppressor role of NME2 has been demonstrated in BCa, lung cancer, oral squamous carcinoma, melanoma, gastric cancer, endometrial cancer, HCC, thyroid carcinoma, and osteosarcoma. As with NME1, NME2 expression is reduced in metastatic tumors compared to non-metastatic tumors and plays a role in cell proliferation, migration, and invasion. Its role is also controversial; we could hypothesize that whether NME promotes or suppresses metastasis depends on the cell type and tumor microenvironment. We focus here on its role as an inhibitor of metastasis and review its role as a promotor of metastasis further on.

As mentioned above, NME1 is a negative regulator of EMT; however, silencing of NME2 did not have any effect on EMT in BCa cells; therefore, NME2 is not involved in the prevention of the earliest stages of metastasis [119]. One study demonstrated that NME1 and NME2 proteins can form mixed oligomers [137]. Nevertheless, knockout mice experiments demonstrated that the loss of NME1 enzyme activity is not compensated by NME2 [18]. However, overexpression of NME2 in squamous cell carcinoma and in gastric cancer decreases metastasis and cell proliferation [138,139], indicating NME2 has metastatic potential.

Some studies have demonstrated that NME2 negatively regulates migration and invasion in cancer, such as BCa, osteosarcoma, gastric, and non-small cell lung cancers. In gastric cancer, NME2 is highly expressed in well-differentiated and less invasive tissue, suggesting a negative correlation between NME2 and cancer progression. Here, NME2 overexpression induces a decrease in cell invasion and cell migration in the collagen matrix [139]. Mechanistically, in BCa, like NME1, NME2 facilitates dynamin 2 (DNM2) oligomerization and increases its GTPase activity, which is required for vesicle scission. NME2-DNM2 interaction may contribute to metastasis suppression by altering tumor cell endocytic and migration phenotypes [130]. As mentioned before, microRNAs could regulate NME2 by inhibiting its expression and, in consequence, inhibit its ability to decrease cell migration. For instance, in osteosarcoma, the level of miR-645 was increased in tumor tissue as compared to normal tissue and could promote cell migration by inhibiting NME2 translation through direct binding to its 3′ untranslated region [140]. In non-small cell lung cancer, circPSMC3 (circular RNA) expression is decreased and is associated with metastasis. Mechanistically, circPSMC3 is a sponge for miR-182-5P, which upregulates NME2 to inhibit migration and invasion [141]. In lung cancer, CARD-recruited membrane-associated protein 3 (CARMA3, which is a member of a family of caspase recruitment domain-containing scaffold proteins and membrane-associated guanylate kinase-like domain-containing proteins) regulates NME2 negatively by NF-κB-dependent induction of mir-182 to promote lung cancer stemness and metastasis [142]. In lung cancer, NME2 regulates focal adhesion function through its binding to the vinculin promoter, inducing a decrease in vinculin expression at the RNA and protein level. Therefore, a decreased expression of NME2 in lung cancer led to enhanced transcription of vinculin and an increase in cell migration and invasion [143]. Only a few studies have focused on the role of NME2 as a protein kinase or histidine kinase in migration or invasion. One study demonstrated that in renal cell carcinoma, MDM2 is able to interact with NME2 and potentially promote the ubiquitylation of NME2 independently of its protein kinase activity; therefore, NME2 is not able to negatively regulate cell migration [144]. However, the role of histidine kinase activity of NME2 in cancer remains to be determined.

The role of NME1/2 in cancer is still controversial and not well-defined in colorectal, gastric, and lung cancers. NME1 expression is associated with a bad prognosis and metastasis in renal and bladder cancers [145], leukemia [146,147], hepatocellular carcinoma [148], squamous cell lung carcinoma [149], ovarian cancer [150], and prostate cancer [151]. It has also been demonstrated that NME1/2 plays a role as a metastasis promoter in neuroblastoma and lymphoma [110].

##### NME1/2 as a Metastasis Promoter

The role of NME1 in HCC is controversial. As mentioned above, NME1 is commonly found to be an inhibitor of metastasis. However, one study demonstrated that NME1 overexpression is associated with a bad prognosis, which is driven by the weak expression of miR146, leading to negative regulation of NME1 expression, resulting in the promotion of cell proliferation [152]. Interestingly, NME1 was first demonstrated as a suppressor of metastasis in melanoma [153]. However, a recent paper showed that NME1 enhances the growth of melanoma spheres in culture, tumor growth, and metastasis to the lung in vivo. It is important to note that NME1 expression in vitro did not affect the proliferation of melanoma cell lines. Therefore, it is important to keep in mind that the role of NME1 could depend on the model of study as well as the tumor microenvironment (TME), which seems to play an important role in whether NME1 suppresses or promotes tumor metastasis [154]. Some studies have suggested the role of NME1 as a protein kinase or histidine kinase in promoting tumor growth and cell proliferation [49,155]. NME1 is highly expressed in esophageal squamous cell carcinoma as compared to normal tissue. Here, NME1 was reported to induce phosphorylation of FAK His58, increasing the proliferation of the cells. However, the direct role of NME1 histidine kinase activity remains to be demonstrated [155]. In liver cancer, pNME1/2 levels are elevated, and there is an increase in 3-pHis proteins, which could play a role in tumor growth [49].

Some studies have demonstrated that NME1 promotes migration and invasion in cancer. One study showed that NME1 RNA and protein expression are associated with increased tumor size and local invasion in colorectal cancer [156,157]. Neuroblastoma is a pediatric cancer associated with aggressive cases with focal amplification on the arm of chromosome 17q of a region that includes NME1 and NME2 located at 17q23. In 21% of neuroblastoma cases, NME1 Ser120 is mutated to a glycine. The S120G mutant NME1 exhibits 50% less phosphotransferase activity compared to the WT recombinant protein. The reduced activity of S120G mutant NME1 is due to the instability of its phosphorylated intermediate, as the His118 phosphate incorporation nor the phosphate transfer from NME1S120G to UDP remain unaffected [72]. Expression of NME1 or NME1-S120G in neuroblastoma cells promotes migration and invasion in culture and metastasis in vivo [92,158]. Due to the genetic landscape of neuroblastoma, it can be postulated that the pro-metastatic role of NME1 is due to an amplification of NME1 expression. Interestingly, silencing NME1 in neuroblastoma increases cell migration [29]. However, this experiment was performed on plastic support, and it is important to recognize, once again, that tumor microenvironment (TME) is important for NME1 to exert its function as a suppressor or promoter of metastasis. Furthermore, in the same paper, the presence of histidine phosphorylation in neuroblastoma cells and tumors was detected on a number of proteins, including NME1 and NME2. Additionally, a set of potential protein targets of NME1 kinase activity with functions in cell adhesion and migration processes has been identified [29]. However, the functional role of histidine kinases and histidine phosphorylation in neuroblastoma tumors remains to be elucidated.

NME2 expression is associated with a bad prognosis in lung cancer [159], HCC, BCa, colorectal carcinoma, neuroblastoma, prostate cancer, gastric cancer, and chronic myeloid leukemia [160] has been reported to inhibit cell migration and invasion. But in contrast to these studies, Boissan et al. demonstrate that NME2 is not a suppressor of EMT, unlike NME1 [119].

A recent study showed that NME2 is upregulated in gastric cancer cell lines, where it maintains the stemness of gastric cancer cells by activating anti-apoptotic genes [161]. Mechanistically, NME2 appears to promote the transcription of antiapoptotic genes, including miR-100 and other survival factors [162].

NME2 promotes proliferation and tumor growth in different cancers, such as HCC [163] and lung and cervical cancers [164]. Mechanistically, NME2 can regulate c-Myc expression, promoting tumor cell proliferation in osteosarcoma cells, Hela cells, and HepG2 cells [165]. In Hela or HepG2 cells, Piwi-like RNA-mediated gene silencing 2 (PIWIL2) can upregulate c-Myc via promoting the binding of NME2 to the c-Myc promoter, inducing tumor cell proliferation. Interestingly, PIWIL2 upregulates RhoA through c-Myc-inducing F-actin filament [166].

Depletion of NME2 in HCC, colorectal carcinoma, or breast cancer cell lines has no impact on invasion or migration [115,167]. NME2 is upregulated in ductal carcinoma in situ and during BCa progression and is highly expressed in invasive tumors. These data point to NME2 not acting as a suppressor of invasion [134]. A few studies demonstrated that NME2 plays a role in resistance to treatment in colorectal cancer and prostate cancer. Depletion of NME2 in CRC cells restored 5-FU sensitivity in 5-FU resistant cells, decreased cell survival, and increased apoptosis [168]. In prostate cancer, NME2 acts as an upstream regulator of MYC, and their increased activity is associated with a risk of resistance to enzalutamide, a treatment in prostate cancer [169]. It has been previously demonstrated that NME2 is able to bind to MYC-promoter region and upregulate MYC transcription [170].

Due to the dichotomous role of NME2 in metastasis, whether NME2 promotes or suppresses metastasis could be dependent on the specific cellular environment, genetic, and tumor microenvironment. Furthermore, NME2’s association with the c-Myc pathway could potentially promote oncogenesis [159] (Table 2).

#### 6.1.2. Histidine Phosphatases

##### PHPT1

To the best of our knowledge, no study has reported a role for PHPT1 as a metastasis suppressor. Rather, most of the studies seem to show that PHPT1 is involved in tumor progression.

High expression of PHPT1 RNA and protein levels appears to be positively correlated with poor prognosis in different cancers, such as HCC [172], lung [173] and pancreatic cancers, and clear cell renal carcinoma [175]. PHPT1 promotes proliferation in HCC [172] and in EGFR mutant lung cancer cells [173]. Mechanistically, in lung cancer, PHPT1 could activate the MAP kinase pathway, which in turn promotes cell proliferation and tumor growth [173]. Furthermore, PHPT1 promotes cell migration and invasion in lung cancer [195]. It has also been shown that PHPT1 is found in filopodia, which are important structures for cell migration [196]. PHPT1 seems to be involved in cytoskeleton reorganization, which is important for cell migration and invasion [196]. PHPT1 plays a role in the resistance to treatment in pancreatic cancer and in the resistance to erlotinib, an EGF receptor inhibitor used in lung cancer treatment [173,174]. All of these studies suggest that PHPT1 acts as a promoter of tumor progression. However, the exact role of PHPT1 as a histidine phosphatase in tumor progression remains to be elucidated.

##### LHPP

LHPP, as mentioned above, is not selective for histidine dephosphorylation; it can also dephosphorylate pLys. Most studies suggest a metastasis suppressor function for LHPP, but LHPP may also play a role as a promoter of tumor progression.

- Metastasis suppressor

LHPP mRNA and protein expression are down-regulated in most cancer tissues, such as bladder urothelial carcinoma, cholangiocarcinoma, colon adenocarcinoma, glioblastoma, kidney renal clear cell carcinoma, paraganglioma, prostate adenocarcinoma, stomach adenocarcinoma [180], and renal cancer [197] and is associated with a poor prognosis. Patients with high LHPP expression have a better prognosis in CRC [181] in brain glioma, renal carcinoma [180], HCC [176], and breast cancer [85].

In HCC, LHPP expression inhibits EMT by targeting TGF-β expression [177]. Furthermore, LHPP regulates tumor progression by inhibiting cell proliferation, migration, and invasion in gastric cancer [180], HCC [177], pancreatic cancer, renal cell carcinoma [197], and bladder cancer [185]. Evidence suggests that LHPP may play a role in mitotic catastrophe, an inherent mechanism for suppressing cancer formation. Mitotic catastrophe is a mechanism of cell death or irreversible growth arrest associated with aberrant mitotic activity. This manifests as multi- and micro-nucleation when mitotic failure occurs due to chromosome breakage, and defects in nuclear division prevent the accumulation of cells with genomic instability [198,199,200]. Here, LHPP inhibits tumor cell growth by promoting mitotic catastrophe through the p27/CyclinA/CDK2 signaling pathway [184] in human esophageal cancer. In nasopharyngeal carcinoma, HDAC4 promotes LHPP deacetylation, enhancing its destabilization. Therefore, LHPP is not able to inhibit TYK2 phosphorylation, activation, and upregulation of STAT1 phosphorylation, thus promoting proliferation and metastasis [201]. Furthermore, LHPP is absent in glioblastoma, and it is associated with a poor prognosis. Here, overexpression of LHPP could impede glycolysis and respiration by inducing ubiquitin-mediated degradation of PKM2, inhibiting the growth of glioblastoma [84].

Another mechanism by which LHPP inhibits metastasis is by downregulating the AKT pathway in various cancers [84,181,185,202,203,204,205,206,207,208,209], thus decreasing cell proliferation, migration, and invasion. In HCC and pancreatic cancer, LHPP binds to the epidermal growth factor receptor (EGFR) and inhibits its phosphorylation, negatively regulating the activation of downstream pathways, including ERK, AKT, and STAT3 [210,211,212], leading to decreased metastatic potential. In cholangiocarcinoma and CRC, LHPP inhibits tumorigenesis by suppressing the TGFβ/Smad signaling pathway [86,213]. In renal cell carcinoma, miR-765, miR-21, and miR-144 can downregulate LHPP expression, promoting EMT, proliferation, and invasion of RCC cells [182].

In gastric cancer (GC), LHPP expression is downregulated in GC tissues and cells that are resistant to cisplatin. The overexpression of LHPP inhibits JNK and p38 MAPK pathways, leading to inhibited stemness and enhanced sensitivity of GC to cisplatin [183].

LHPP acts as a tumor suppressor in liver cancer. Its expression is downregulated (in contrast to NME1/2, whose expression is upregulated), correlating with an increased level of 1- and 3-pHis present on proteins [49]. However, the precise molecular mechanism by which LHPP functions as a metastasis suppressor via its histidine phosphatase activity in cancer remains unexplored. One recent study demonstrated that in gastric cancer, LHPP overexpression induces a decrease in 3-pHis labeling [202]. It was suggested that the specific enzymatic active site of LHPP is the cysteine residues at positions 53 and 226 [47]. However, when mutant LHPP is overexpressed in GC cells, pHis levels decrease as compared to wild-type LHPP, indicating that these residues may not be involved in the catalytic activity of LHPP [202]. LHPP is a member of the HAD family of phosphatase, so we can postulate that the catalytic activity of LHPP is mediated by its two essential catalytic Asp residues.

- Metastasis promotor

There are not many studies about the role of LHPP as a promoter of metastasis. One study found that LHPP is associated with hyperthyroidism, and overexpression of LHPP in hyperthyroidism may contribute to carcinogenesis in the thyroid [178]. Furthermore, another study showed that siRNA knockdown of LHPP inhibits cell growth and migration of highly aggressive metastatic BCa cells [179]. Our lab also demonstrated that LHPP knockdown in TNBC cells reduces migration in culture and metastasis in mice with MDA-MB-231 flank xenograft tumors, confirming its role as a metastasis promoter [85]. Therefore, the role of LHPP could vary depending on tissue type and cellular context, potentially holding significant relevance in cancer progression.

##### PGAM5

To the best of our knowledge, there is no study that reports on the role of PGAM5 acting as a metastasis suppressor. Rather, most of the studies seem to suggest that PGAM5 is a promoter of tumor progression.

PGAM5 overexpression at the RNA and protein level is associated with a bad prognosis in prostate cancer [194], lung adenocarcinoma [190], HCC [186,187,188,214], colon cancer [193], melanoma [191], and non-small cell lung cancer [192].

PGAM5 is well known to play a role in mitophagy, the process of selective degradation of mitochondria that are damaged or redundant. PGAM5 was shown to regulate PINK1/Parkin-mediated mitophagy independently of its phosphatase activity. In HCC, PGAM5 depletion reduced tumor growth through a decrease in mitophagy, enhanced apoptosis, and increased ATP production [186,187,188,214]. Likewise, Staphylococcal nuclease domain-containing protein 1 (Snd1) localizes to the mitochondria and promotes PGAM5-mediated mitophagy and liver cancer progression [215]. In CRC, expression of PGAM5 proteins and the mitophagy-related protein parkin are elevated in tumor tissue and correlate with advanced CRC. Here, PGAM5 activates serine/threonine PTEN-induced putative kinase1(PINK1)/parkin pathway-mediated autophagy [193]. Furthermore, S100 calcium-binding protein A9 (S100A9) promotes binding between ubiquitin-specific peptidase 10 (USP10) and PGAM5, leading to the deubiquitylation and stabilization of PGAM5, which increases mitochondrial ROS levels by promoting mitochondrial fission and inducing the growth and metastasis of HCC [216]. Another mechanism by which PGAM5 could promote tumorigenesis in gastric cancer is through PI3K/AKT pathways [217].

In HCC, high PGAM5 expression induces chemoresistance to 5-fluorouracil. Mechanistically, PGAM5 inhibits BAX- and cytochrome C-mediated apoptotic signaling and then enhances the Bcl-xL-mediated anti-apoptotic pathway, which is associated with a poor prognosis [206]. Few studies demonstrate the role of PGAM5 phosphatase activity in liver cancer and CRC. In liver cancer, the deacetylase SIRT2 deacetylates the lysine K191 site of PGAM5, stimulating the protein phosphatase activity of PGAM5. PGAM5, in turn, dephosphorylates and activates malic enzyme 1 (ME1), leading to lipid accumulation and liver cancer cell proliferation [189]. In CRC, it has been shown that PGAM5 is upregulated and dephosphorylated ME1 at S336, leading to ME1 dimerization and activation, promoting tumorigenesis. Interestingly, in liver cancer, PGAM5 dephosphorylates the total level of ME1 without altering the dephosphorylation of S336 ME1 [218].

### 6.2. NME1/2 and Phosphatase in Other Biological Processes

#### 6.2.1. NME1/2

NME1/2 and the catalytic H118 residue are highly conserved across species, spanning from yeast to humans, and have been shown to have important functions in development and other biological processes [219,220,221]. In mice, single knockouts of NME1 or NME2 are viable, but double knockouts are lethal perinatally [18,19,21]. Additionally, NME1 knockout female mice display poor mammary duct development [17], while NME2 is implicated in erythroid lineage development, evident from abnormalities in erythrocyte maturation in Nme2 knockout mouse embryos [21]. To investigate the role of histidine phosphorylation in specific functions, knock-in mice deficient for NME NDPK and protein–histidine kinase activities should be generated. In *C. elegans*, NME1 plays an important role in gonadal development [222]. Moreover, phosphorylation of H266 on the Gβ subunit by NME2 is a key intermediate in the activation of G protein signaling, which is needed for the regulation of cardiac contractility [68,223,224]. Likewise, in zebrafish, NME2 knockdown induces a decrease in cardiac contractility and vessel formation [225].

Additional studies have shown the importance of NME1/2 in other biological contexts. As mentioned earlier, NME1 and NME2, via their NDPK function, play a role in the endocytosis of several receptors and, through this interaction, inhibit cell migration [130,226,227]. NME1 and NME2 are also DNA-binding proteins [228], with NME1 promoting DNA damage repair induced by ultraviolet radiation [229,230], and their NDPK activities seem to play a role in DNA repair [231,232]. It has also been demonstrated that NME1 promotes genomic stability by modulating the following DNA repair pathways: homologous recombination (HR), non-homologous end joining (NHEJ) of double-strand break repair (DSBR) and alternative NHEJ, nucleotide excision repair (NER), and double-strand break repair [231]. Moreover, NME1/2’s interaction with the CFTR (cystic fibrosis transmembrane conductance regulator) implicates their involvement in cystic fibrosis [233,234]. Here, the NDPK phosphotransferase function of NME1 is required for the AMPK-dependent inhibition of CFTR. NME2 plays a role in glucose metabolism [235] through its NDPK activity and the association of the NME1/prune complex with neurodevelopmental [236] disorders, underscoring their impact in disease contexts. The NDPK activity of NME has been studied in relation to biological development and disease. However, understanding the role of histidine phosphorylation mediated by NME1/2 in these processes is therefore crucial for unraveling their biological significance.

#### 6.2.2. PHPT1

Srivastava et al. [67] successfully produced *phpt1*^−/−^ mice, which showed increased levels of perinatal mortality compared to normal mice. When analyzing blood samples, it was discovered that newborn *phpt1*^−/−^ mice had reduced levels of blood glucose and increased serum insulin, but within 2–3 days post-birth, glucose levels returned to normal. Islets isolated from *phpt1*^−/−^ mice pancreas revealed increased Ca^2+^ and lower insulin levels when cultured under low glucose conditions compared to normal mice. Finally, after a glucose tolerance test, *phpt1*^−/−^ mice showed lower levels of serum insulin and higher levels of blood glucose. The proposed mechanism is that activation of the TRPC4 calcium channel by PHPT1 leads to increased Ca^2+^ levels inside the cell, which activates the CAMKKB protein kinase. This, in turn, leads to the activation of AMPK and increased trafficking of KATP, a potassium channel involved in insulin secretion, to the plasma membrane.

PHPT1 has also been implicated in controlling CD4^+^ activation [65]. It has been shown that phosphorylation of His358 in the KCa3.1 calcium-activated ion channel (a target residue of PHPT1) is required for reactivation of CD4 T cells [63]. Upon siRNA knockdown of PHPT1, primary human CD4^+^ T cells required a 10-fold lower concentration of dendritic cells (pulsed with staphylococcal enterotoxin B) for activation.

After the initial discovery of PHPT1, a report from Klumpp et al. [39] found that the *C. elegans* ortholog of PHPT1, PHIP-1, was almost entirely localized in neural tissue. Although no biological role was proposed in this study, Sakai et al. [69] recently demonstrated that PHIP-1 plays a critical role in axon regeneration. Neurons that had PHIP-1 knocked out via CRISPR/Cas9 or had a point mutation changing the catalytic histidine to alanine, thus rendering the protein inactive, displayed impaired neuron regeneration, which could be reversed upon re-expression of wild-type PHIP-1. Overexpression of NDK-1, the ortholog of NME1/2, also inhibited neuron regeneration, suggesting these two proteins are affecting the same pathway. To identify potential targets of PHPT1, the authors performed a yeast two-hybrid screen and identified the C. elegans homologs of Gβ (GBP-1) and GAPDH (GPD-2/3/4). Further testing revealed GPB-1 His266 as the target residue, and phosphorylation of this residue could activate GOA-1 (Goα ortholog), leading to repressed axon regeneration.

A publication by Oh et al. [237] investigated brown adipocyte differentiation to determine if pHis may play a role in this process. Using immortalized preadipocyte cells, they found that pHis levels decreased in most proteins 2 days after differentiation initiation, and after 6 days, pHis levels returned to control levels. This same result was found for PHPT1 protein levels, with a strong decrease during day 2 and subsequent recovery. Cells with stably expressed PHPT1 shRNA were found to have lower mRNA levels for proteins associated with brown adipocytes, including PGC1α, PRDM16, PPARγ, and UCP1, as well as increased lipid droplet formation after 6 days of differentiation, while cells that stably overexpressed PHPT1 showed the opposite effects. Although a mechanism for this effect has not been reported, it is possible that ACLY may be involved, as it is both a target of PHPT1 phosphatase activity and involved in fatty acid metabolism. In line with this, the authors found that ACLY pHis levels were increased upon knockdown of PHPT1 while the opposite occurred in PHPT1 overexpressing cells.

One of the functions for which PHPT1 has been implicated is in cell migration. Xu et al. [238] found that after the addition of PDGF to induce lamellipodia formation, both PHPT1, Arp3 (involved in actin filament nucleation), and F-actin colocalized to the leading edge of lamellipodia in HuH-7 cells while knockdown of shRNA PHPT1 reduced Arp3 colocalization. A PHPT1 pulldown experiment did not detect a PHPT1-Arp3 interaction, but an in vitro F-actin binding assay showed that PHPT1 can bind to F-actin. In another report [239], the same group also reported that PHPT1 levels were elevated in fibrotic liver tissue compared to normal tissue. Staining of liver samples showed that this upregulation primarily took place in hepatic stellate cells (HSC), which was confirmed by immunoblotting of isolated mouse HSC. Treatment of the human HSC cell line LX-2 with TGF-β1 showed a dramatic increase in PHPT1 protein levels in a dose-dependent manner, although interestingly, PHPT1 mRNA levels did not significantly change. The use of the TGF-β1 receptor inhibitor SB525334 was able to block this increase. Migration assays revealed that migration was dependent on PHPT1 levels, with overexpressing cells migrating more while shRNA-treated cells migrated less. Analysis of downstream pathways suggested that these effects are being mediated through the PI3Kγ/AKT/Rac1 pathway. Using a CCl_4_-induced liver fibrosis mouse model, the knockdown of PHPT1 resulted in reduced recruitment of macrophages to the wound site [240]. This is likely due to the reduced mobility of these immune cells, as evidenced by reduced podosome/invadosome formation and inhibition of Akt pathway activation.

Several studies have noted that transcription of *phpt1* is greatly upregulated after exposure to ionizing radiation. An early study by Dressman et al. [241] performed gene expression analysis on human peripheral blood samples after exposure to 150 cGy–1350 cGy of radiation to identify changes in expression, which would indicate exposure to ionizing radiation. One of the genes identified was *phpt1*. Follow-up studies [242,243] found that *phpt1* mRNA could be upregulated after exposure to 50 mGy of radiation in as few as 6 h [242] and as long as 48 h post-exposure [244], with up to a 12-fold increase in mRNA levels. While intriguing, no functional role for PHPT1 in radiation exposure has been proposed thus far.

#### 6.2.3. LHPP

According to GWAS studies, LHPP has also been associated with major depressive disorders [245,246,247], alcohol dependence, and risky behavior [248].

Recently, one study demonstrated that *lhpp* knockout mice display no observable phenotype but exhibit resilience to depression-like behaviors induced by chronic stress. Specifically, LHPP exhibits its activity at acidic pH, which is expressed in the lysosome of astrocytes. Under stress conditions, LHPP regulates lysosomal acidification through pyrophosphate (PP_i_) hydrolysis-driven vacuolar ATPase (V-ATPase)-dependent proton transport. LHPP deficiency impedes lysosomal dependent-degradation of the C/EBPβ transcription factor, leading to the expression of a set of chemokines that ameliorate the inhibitory effect of chronic stress and promote adult neurogenesis [249]. However, LHPP’s role as a phosphatase in biological development remains to be elucidated.

#### 6.2.4. PGAM5

PGAM5 is known to play a role in the brain. PGAM5 KO mice show Parkinson-like movement [250]. Here, PGAM5 is required for the stabilization of the mitophagy-inducing protein PINK1 on damaged mitochondria. Loss of PGAM5 disables PINK-1-mediated mitophagy and leads to dopaminergic neurodegeneration and dopamine loss. Additionally, PGAM5 plays a role in necroptosis. Autoimmune hepatitis (AIH) is a severe necrotic inflammatory liver disease associated with significant mortality. PGAM5 is highly expressed in the hepatocytes of patients with AIH. PGAM5 KO mice are resistant to concanavalin A (ConA), which is used to induce hepatocellular death and liver injury. Here, PGAM5 plays a role in the pathogenesis of ConA-induced liver injury. Mechanistically dynamin-related protein 1 (Drp1)-mediated mitochondrial fission is downstream of PGAM5, promoting hepatic necrosis and liver injury [251].

Little is currently known about the role of PGAM5 as a phosphatase during biological development or disease. One paper has shown that PGAM5 plays a role in metabolic stress. Here, PGAM5 KO mice are resistant to severe metabolic stress and show suppressed lipid accumulation. PGAM5 appears to play a role in the lipid accumulation process. PGAM5-KO brown adipocytes have an increased oxygen consumption rate and expression of uncoupling protein 1 (UCP1), which enhances energy consumption in mitochondria. In this paper, it was shown that PGAM 5 phosphatase activity and intramembrane cleavage are necessary to inhibit UCP1 activity [252].

An additional role for PGAM5 as a histidine phosphatase is its role in controlling CD4^+^ T cell activation. Panda et al. [51] found that PGAM5 can dephosphorylate His118 on NME2, resulting in decreased phosphorylation of the KCa3.1 ion channel, which has been shown to decrease cell activation [65]. *pgam5*^−/−^ knockout mice were found to be viable but displayed Parkinson-like movement disorder around 1 year of age. Knockdown of PGAM5 via siRNA in Jurkat T cells resulted in increased Ca2+ influx and pro-inflammatory cytokine production upon TCR stimulation. CD4^+^ T cells isolated from *pgam5*^−/−^ mice showed similar results, along with increased levels of pHis on both NME2 and KCa3.1. Allogenic transfer of CD4^+^ T cells from *pgam5*^−/−^ mice into normal mice resulted in increased INF-γ and TNF-α levels, significantly lower rates of survival, and more severe graft versus host disease pathologies.

## 7. Methods to Detect Histidine Phosphorylation in Cells and Tissue

### 7.1. Antibodies That Recognize pHis

Due to their immaculate specificity in recognizing antigens, antibodies are essential tools for research and therapeutic development. However, the unstable nature of the P-N bond within pHis under physiological conditions hindered earlier efforts to generate antibodies against this modification. Advances in the synthesis of non-hydrolyzable pHis analogs provided plausible routes to utilizing them to raise antibodies against pHis. Consequently, different groups developed pHis antibodies using analogues like thiophosphohistidine, phosphofurylalanine, phosphopyrrolylalanine, 4-phosphothiophen-2-yl-alanine, phosphoryl-triazolylethylamine (pTze), 1-phosphotriazolylalanine (1-pTza), 3-phosphotriazolylalanine (3pTza), phosphono-pyrazolyl ethylamine (pPye), and 4-phosphopyrazol-2-yl alanine (pPza) [253,254,255,256,257,258,259,260]. The Muir and Webb research groups developed stable phosphoryltriazolylalanine (Tza) analogs of 1-pHis and 3-pHis, enabling the creation of antibodies specific to these pHis isoforms [257,258]. Initial efforts led to the development of a 3-pHis antibody against histone H4 pH18, followed by sequence-independent antibodies towards 3-pHis [257]. Later, McAllister et al. designed additional triazole phosphohistidine analogs that facilitated antibody production to detect pHis. However, these early efforts yielded pHis antibodies that suffered either significant crossreactivity to pTyr or reduced affinity to native pHis modification [15]. The most notable pHis antibodies were developed by Fuhs et al. using 1-pTza and 3-pTza analogs embedded in degenerate libraries of alanine and glycine peptides [15]. Antibodies SC1-1 and SC50-3 were found to recognize 1-pHis, while SC39-4, SC44-8 and SC56-2 recognize 3-pHis modifications. They have unique properties of isomer specificity, meaning they do not crossreact with the other isoforms of pHis and pTyr, allowing for use by many research groups in immunoblotting, immunofluorescence staining, immunohistochemistry, and co-immunoprecipitation experiments. Structures of these pHis mAbs in complex with pTza peptides ligands provided insight into the mode of phosphate recognition, the distinguishing features of the paratope that determines isomer specificity, and the structural basis for the lack of crossreactivity to other phosphoamino acids [2]. Furthermore, this structural information laid the groundwork for antibody engineering to improve their affinity and specificity towards a wide range of pHis substrates. Contrary to conventional stereochemistry-based predictions, Makwana et al. employed density functional theory (DFT) calculations to propose pyrazolyl ethylamine and pyridine amino amide as optimal analogs of 3-pHis and 1-pHis, respectively. Subsequently, these analogs were used to develop 1- and 3-pHis polyclonal antibodies in sheep, which were shown to be effective in biochemical experiments [261].

With the development of 1-pHis monoclonal antibodies by Fuhs et al., pNME1/2 levels could be detected by immunoblotting, which was used to demonstrate elevated 1-pHis levels in NME1/2 in hepatocellular carcinoma and neuroblastoma [29,49]. Furthermore, the first sequence-dependent anti-pH118 NME1/2 polyclonal antibody was developed, enabling the detection of pNME H118 levels in various cell lines [29,49,219]. With the development of 1-pHis or 3-pHis rabbit monoclonal antibodies, detection of pHis was achieved through various methods, including by immunoblotting in different cell lines [15,29,49], immunofluorescence staining in neuroblastoma [29] and HeLa cells, [15] and immunohistochemistry in pancreatic cancer [262] by modifying and optimizing the protocols to conserve pHis signals during sample preparation [263,264,265].

### 7.2. Mass Spectrometry

Mass spectrometry (MS) is a critical tool in the analysis of the phosphoproteomes of pSer, pThr, and pTyr modifications. However, its usefulness in identifying pHis phosphosites is still under scrutiny. In positive ion mode, MS analysis utilizes acidic conditions to enrich and HPLC fractionate phosphopeptides; thus, conserving the pHis signal under these conditions is a challenge. However, efforts are being invested in finding alternatives to the conventional methods of enrichment. The use of Fe^+2^-IMAC and TiO_2_ enrichment methods at reduced acidic conditions uncovered 11.6% and 6.3% of pHis phosphosites in *E. coli* and zebrafish larvae [266,267]. A new method called UPAX, which combines strong anion exchange chromatography at pH 6.8 with tandem MS/MS, was developed to enrich pHis peptides along with other noncanonical phosphosites like pArg and pLys from HeLa cells [267]. This study attributed 30% of the phosphoproteome to the noncanonical phosphosites, of which pHis is harbored in 6% of the sites. Another enrichment method uses sub-2 μm core-shell silica microspheres containing bis(zinc(II)-dipicolylamine) adducts to enrich pHis peptides under neutral conditions, resulting in recovering 19 and 611 pHis sites from *E. coli* and HeLa cells, respectively [268]. An alternate pHis peptide enrichment method reported by Adam et al. used hydroxyapatite chromatography followed by immunoaffinity enrichment of pHis peptides using immobilized antibodies against 1-pHis and 3-pHis followed by MS/MS analysis, leading to the identification of 77 HeLa cell pHis sites, although the majority of these identified proteins have yet to be fully validated [269]. While mass spectrometry is a promising technique for identifying novel pHis sites in proteins, further improvements in the sample preparation steps of the MS workflow are needed, as current methods lead to significant loss of pHis signal [270]. An alternative to MS workflow was developed by Makwana et al., where 31P NMR spectroscopic analysis was used to quantify the levels of phosphohistidine levels in the human bronchial epithelial cells [270]. By avoiding tryptic digestion and acidic pH, this method reported pHis to be the third largest PTM after pSer and pThr and 15 times more abundant than pTyr modification. Though functional characterization of each of the individual sites uncovered in these studies has still not been undertaken, there are reports that strike down the authenticity of the pHis sites. Leijten et al. developed a stringent workflow to analyze pHis sites and reported that the widely assigned pHis sites in mammalian systems should be site localized to either Ser or Thr and wrongly assigned to His and argue that the pHis is negligent in mammalian cells [271].

### 7.3. Small Molecule Activity Modulators of Histidine Kinases and Phosphatases

Small molecule activity modulators, whether altering the catalytic activity of an enzyme or disrupting/stabilizing a protein–protein interaction (PPI), are invaluable tools for studying the functional role of a given protein. While CRISPR/Cas9 knockout and siRNA knockdown have proven to be invaluable in this regard, their use results in the absence of the protein from the cell. This can have unforeseen side effects, such as activation of pathways due to missing PPIs or compensatory mechanisms to overcome the missing protein. Activity modulators do not have these drawbacks and can allow for the study of a protein in a more natural context. As such, the development of these small molecule compounds is of great interest for studying the functional role of pHis in cells.

#### 7.3.1. PHPT1

The first PHPT1 inhibitors were identified in a doctoral thesis by Eerland [272], where several sulfonamide imidazole compounds were prepared and identified as inhibitors of PHPT1 in vitro. These compounds were found to have IC_50_ values ranging from 11 µM to 3 µM, with the most potent compound having modest cell permeability. Unfortunately, in a patch clamp assay to monitor the activity of the KCa3.1 ion channel, the addition of 10 µM of the compounds did not increase the activity of the channel, which would be expected given that PHPT1 has been shown to decrease KCa3.1 channel activity. Furthermore, activation of the KCa3.1 channel by the activator DCEBIO was inhibited upon the addition of the compound, suggesting possible off-target effects.

The next identified inhibitors were discussed in McCullough et al. [273], where a 2000 compound high-throughput screen found four compounds: roxarsone and acetarsol, both aromatic arsonic acids, and stictic acid and norstictic acid, natural products derived from lichen. Characterization of these compounds revealed that inhibition of the arsonic acids was reversible, competitive inhibitors with IC_50_ values around 100 µM, while the natural products were modestly potent irreversible inhibitors with a K_I_ of 90 µM and k_inact_ of 1.7 min^−1^.

Inspired by the structural characteristics of norstictic acid, Wang et al. [274] explored another natural product, illudalic acid, to determine if it could act as a PHPT1 inhibitor. Using both the parent compound as well as several synthetic derivatives, dose–response testing found that most of these compounds had IC_50_ values in the single-digit micromolar range. However, unlike norstictic acid, these were found to be reversible, non-competitive inhibitors.

A final report by Kim et al. [275] describes a 9000 compound screen that identified ethacrynic acid as a PHPT1 inhibitor. This compound was found to be an irreversible inhibitor with *K*_I_ of 183 µM and k_inact_ of 2.67 min^−1^. An investigation into the binding site revealed that the compound targets a highly conserved Cys residue located near the active site pocket of PHPT1. Interestingly, mutation of this cysteine and two proximal cysteine residues did not impact PHPT1 activity [276], suggesting the inhibition observed upon compound binding may be due to disruption of the enzyme structure or blocking a conformation required for catalysis.

#### 7.3.2. PGAM5

Although no compounds have been tested against its histidine phosphatase activity, a couple of compounds capable of inhibiting PGAM5 enzyme activity have been reported. Gao et al. [277] investigated a compound called LFHP-1c, which had been previously shown to provide a neuroprotective role in ischemia. Attempting to elucidate a mechanism of action, they performed a capture experiment in which lysate was incubated with an immobilized compound. PGAM5 was identified as one of the hits, and follow-up surface plasmon resonance experiments confirmed this binding with a K_d_ of 961 nM. The compound could reduce PGAM5 activity in isolated mitochondria and was reported to decrease enzyme activity in a dose-dependent manner, although the substrate used is not clear. Using a modified photoaffinity crosslinking variant of LFHP-1c, the authors were able to confirm that the compound binds to PGAM5 directly in cells.

Wilkins et al. [56] were able to identify a sequence in PGAM5 that is necessary for the assembly of the enzyme into large multimeric complexes. This motif, WDXNWD, was shown to be required for PGAM5 phosphatase activity, and alanine mutants within this region, which should result in disruption of multimer formation, showed reduced phosphatase activity. A small peptide consisting of residues 52–74, which contains the WDXNWD motif, was found to inhibit PGAM5 activity towards phosphoserine peptides in a dose-dependent manner, although enzyme activity was never fully eliminated. This inhibition was found to occur in a non-competitive manner, consistent with the proposed allosteric mechanism of action.

#### 7.3.3. NME1/2

To date, there are currently no well-characterized small molecule inhibitors of the NME1/2 enzymes. A report by Malmquist et al. [278] identified ellagic acid as an inhibitor of NME2 with an IC_50_ value of 23 µM. However, the activity assay was performed using cell lysate with a luciferin–luciferase ATP assay rather than purified protein, so it is unclear if the observed reduction in activity is solely resulting from NME2 inhibition. More recently, Mortenson et al. [279] studied the binding of small molecule electrophiles to various proteins. One of the targets used in this study was NME1, which was found to bind one of the probes tested. When a competition binding assay with ellagic acid was performed, the binding of the probe was reduced, suggesting that these two compounds may bind to the same region on the protein. Given this, it is possible that the probe (or a similar compound) might act as an inhibitor, but no data have been reported thus far. Further studies to characterize these compounds would be of interest, as there is currently an unmet need for active site-targeting inhibitors of these two enzymes.

While reducing enzyme activity is helpful in identifying a given target’s cellular function, increasing enzyme activity can be equally helpful in certain situations. Lee et al. [280] discovered a small molecule activator of NME1 called NMac1, which increased enzyme activity through a reduction in substrate *K*_m_, which the authors propose occurs through an allosteric binding site. In the treatment of hexameric, but not dimeric, NME1 was found to increase enzyme activity, and NMR hydrogen/deuterium exchange experiments suggest that binding occurs on residues 142–152. The addition of NMac1 to MDA-MB-231 cells showed decreased invasion and migration in a dose-dependent manner, and injection of these cells into SCID mice found that the treatment cohort, which received 10 mg/kg NMac1, showed significantly reduced breast cancer metastasis. It is currently not known if NMac1 is capable of modulating pHis levels within a cell.

## 8. Conclusions

Histidine phosphorylation represents a crucial yet underexplored post-translational modification with significant implications for cellular function and regulatory mechanisms. The distinct physicochemical properties of phosphohistidine, including its acid-lability and thermal instability, have historically hampered its study. However, recent advances, particularly the development of specific antibodies and novel analytical techniques, have begun to unravel the complexity of histidine phosphorylation. Key players such as NME1, NME2, PHPT1, LHPP, and PGAM5 have been identified as central to the dynamic regulation of this modification, influencing a wide array of cellular processes ranging from ion channel activity to metabolic enzyme function and signal transduction pathways. Moreover, the emerging evidence linking histidine phosphorylation to cancer progression underscores its potential as a therapeutic target. While considerable progress has been made, many aspects of histidine phosphorylation, including its full spectrum of substrates and regulatory mechanisms, remain to be elucidated. Future research should focus on developing more robust and sensitive detection methods and investigating their role in diverse physiological and pathological contexts. This will not only enhance our understanding of cellular regulation but also pave the way for novel therapeutic interventions targeting phosphohistidine pathways.

## Figures and Tables

**Table 2:** Summary of anti-tumor and tumor promotion effect of His kinases and phosphatases on different cancers.

	Anti-Tumoral		Pro-Tumoral	
Kinases	Cancer	Kinase Activity	Cancer	Kinase Activity
**NME1**	Breast cancer [115,125,133]	Decreases proliferation. Functions as a histidine kinase that phosphorylates KSR S392 [81].	HCC [49,152]	Increases proliferation. Functions as a histidine kinase. Increase in pNME1/2 and pHis proteins [49].
		Decreases migration. Functions as an NDPK by generating GTP for dynamin [130,131].	Esophageal squamous cell carcinoma [155]	Increases proliferation. Functions as a histidine kinase by phosphorylation of FAK His58 [155].
		Decreases proliferation. Functions as a histidine kinase [30].	Neuroblastoma [72,92,110,158]	Functions as a histidine kinase. Increase in pNME1/2 and pHis proteins [29].
	Relation to kinase activity unknown: ovarian cancer [108], hepatoma [115], CRC [115,124], melanoma [108], HCC [104,115,133], gastric cancer [116], glioma [117], nasopharyngeal carcinoma [118], and lung cancer [120].		Relation to kinase activity unknown: melanoma [154], CRC [156,157], and ovarian cancer [171].	
**NME2**	Breast cancer [130]	Decreases migration. Functions as an NDPK and generates GTP for dynamin [130].	Neuroblastoma [72]	Increase in pNME1/2 and pHis proteins [72].
	Relation to kinase activity unknown: lung cancer [142,143], renal cell carcinoma [144], gastric cancer [139], osteosarcoma [140].		Relation to kinase activity unknown: HCC [163], breast cancer [134], colorectal carcinoma [168], lung cancer [164], prostate cancer [169], gastric cancer [161], chronic myeloid leukemia [160], cervical cancer, [164] and osteosarcoma cells [165].	
**Phosphatases**	**Cancer**	**Phosphatase activity**	**Cancer**	**Phosphatase activity**
**PHPT1**			Relation to phosphatase activity unknown: HCC [172], lung cancer [173], pancreatic cancer [174], and clear cell renal carcinoma [175].	
**LHPP**	HCC [176,177]	Affects tumor growth. Functions as a phosphatase. Decrease in 3pHis [49].	Relation to phosphatase activity unknown: thyroid carcinogenesis [178] and breast cancer [179].	
	Relation to phosphatase activity unknown: urothelial carcinoma [180], cholangiocarcinoma [180], colon adenocarcinoma [180], paraganglioma [180], prostate adenocarcinoma [180], stomach adenocarcinoma [180], colorectal carcinoma [181], pancreatic cancer, renal cell carcinoma [182], gastric cancer [180,183], nasopharyngeal carcinoma, esophageal cancer [184], glioblastoma, oral squamous cell carcinoma, prostate cancer, papillary thyroid cancer, cervical cancer, and bladder cancer [185].			
**PGAM5**			Liver cancer [186,187,188,189]	Increases cell proliferation. Functions as a phosphatase. Dephosphorylates ME1 (Malic Enzyme 1) [189].
			Relation to phosphatase activity unknown: lung adenocarcinoma [190], melanoma [191], lung cancer [192], colorectal carcinoma [193], prostate cancer [194].	

## Abbreviations

NDPK	Nucleoside diphosphate kinase
NTPs	Nucleoside triphosphates
NDPs	Nucleoside diphosphates
His Kinase	Phosphohistidine kinase
TCS	Two-component system
Coenzyme A	CoA
KSR	Kinase suppressor of Ras
MSS	Metastasis Suppressor Signature
MLC	Myosin light chain
pNME	Phosphorylated NME
PGAM1	Phosphoglycerate mutase 1
CAMKII	Calcium/calmodulin-dependent kinase II
TRPV5	Transient receptor potential cation channel subfamily V member 5
GNB1	Guanine nucleotide-binding protein G(I)/G(S)/G(T) subunit beta-1
KCa3.1	Intermediate conductance calcium-activated potassium channel protein 4
ACLY	ATP-citrate synthase
SCSα	Succinyl-CoA synthetase alpha
KSR1	Kinase suppressor of Ras 1
ANXA1	Annexin A1
ALDOC	Aldolase C
PGAM5	Phosphoglycerate mutase 5
LCFA-CoA	Long-chain fatty acyl-CoA
PHPT1	Phosphohistidine phosphatase 1
LHPP	Phospholysine phosphohistidine inorganic pyrophosphate phosphatase
